# Self-redox reaction driven in situ formation of Cu_2_O/Ti_3_C_2_T_x_ nanosheets boost the photocatalytic eradication of multi-drug resistant bacteria from infected wound

**DOI:** 10.1186/s12951-022-01428-3

**Published:** 2022-05-19

**Authors:** Ya-Ju Hsu, Amit Nain, Yu-Feng Lin, Yu-Ting Tseng, Yu-Jia Li, Arumugam Sangili, Pavitra Srivastava, Hui-Ling Yu, Yu-Fen Huang, Chih-Ching Huang, Huan-Tsung Chang

**Affiliations:** 1 Department of Chemistry, National Taiwan University, Taipei, 10617 Taiwan; 2 School of Basic Sciences, Indian Institute of Technology, Kamand campus, Mandi, Himachal Pradesh 175005 India; 3 Institute of Analytical and Environmental Sciences, National Tsing Hua University, Hsinchu, 30013 Taiwan; 4 Department of Bioscience and Biotechnology and Center of Excellence for the Oceans, National Taiwan Ocean University, Keelung, 202301 Taiwan; 5 School of Pharmacy, College of Pharmacy, Kaohsiung Medical University, Kaohsiung, 80708 Taiwan

**Keywords:** MXene, Cuprous oxide, Photoresponsive nanomaterials, Antimicrobials, Wound healing

## Abstract

**Background:**

MXenes with interesting optical and electrical properties have been attractive in biomedical applications such as antibacterial and anticancer agents, but their low photogeneration efficiency of reactive oxygen species (ROS) and poor stability are major concerns against microbial resistance.

**Methods:**

Water-dispersible single layer Ti_3_C_2_T_x_-based MXene through etching tightly stacked MAX phase precursor using a minimally intensive layer delamination method. After addition of Cu(II) ions, the adsorbed Cu(II) ions underwent self-redox reactions with the surface oxygenated moieties of MXene, leading to in situ formation of Cu_2_O species to yield Cu_2_O/Ti_3_C_2_T_x_ nanosheets (heterostructures).

**Results:**

Under NIR irradiation, the Cu_2_O enhanced generation of electron–hole pairs, which boosted the photocatalytic production of superoxide and subsequent transformation into hydrogen peroxide. Broad-spectrum antimicrobial performance of Cu_2_O/Ti_3_C_2_T_x_ nanosheets with sharp edges is attributed to the direct contact-induced membrane disruption, localized photothermal therapy, and in situ generated cytotoxic free radicals. The minimum inhibitory concentration of Cu_2_O/Ti_3_C_2_T_x_ nanosheets reduced at least tenfold upon NIR laser irradiation compared to pristine Cu_2_O/Ti_3_C_2_T_x_ nanosheets. The Cu_2_O/Ti_3_C_2_T_x_ nanosheets were topically administrated on the methicillin-resistant *Staphylococcus aureus* (MRSA) infected wounds on diabetic mice.

**Conclusion:**

Upon NIR illumination, Cu_2_O/Ti_3_C_2_T_x_ nanosheets eradicated MRSA and their associated biofilm to promote wound healing. The Cu_2_O/Ti_3_C_2_T_x_ nanosheets with superior catalytic and photothermal properties have a great scope as an effective antimicrobial modality for the treatment of infected wounds.

**Graphical Abstract:**

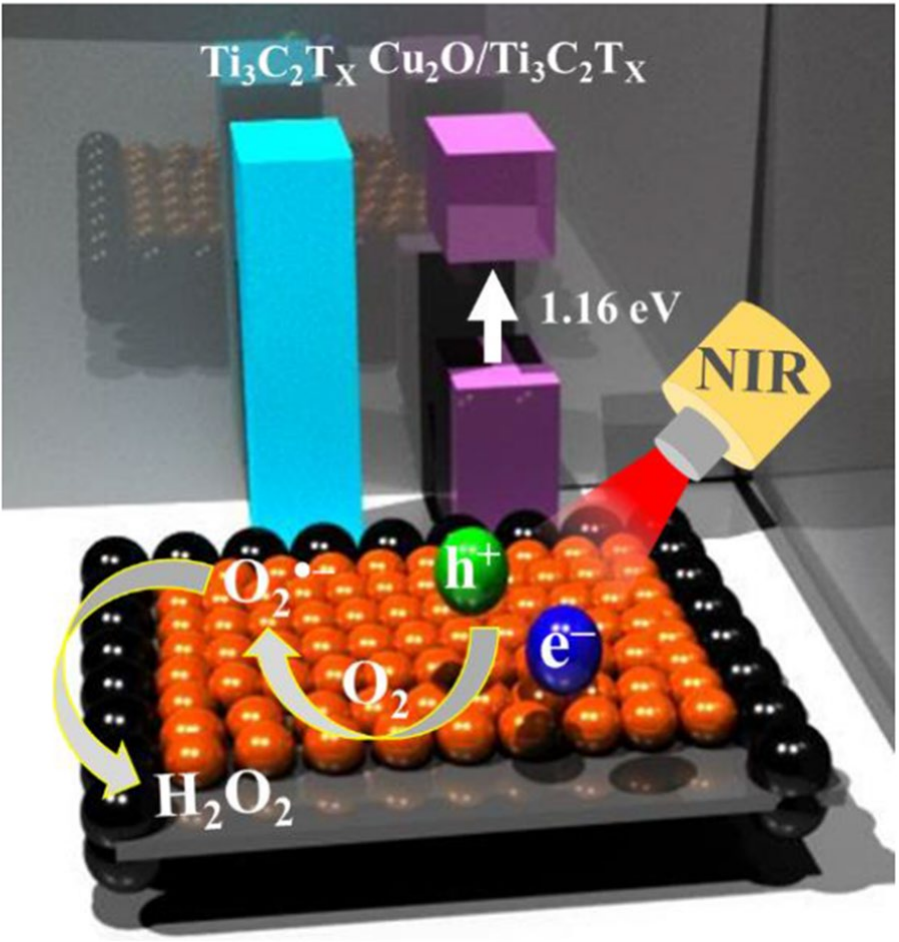

**Supplementary Information:**

The online version contains supplementary material available at 10.1186/s12951-022-01428-3.

## Background

Infectious diseases pose a serious threat to humankind and global economy [1]. Antimicrobial resistance (AMR), gave rise to the situation of global health emergency [2]. Once bacteria come in contact with an exposed wound, they consume nutrients from surrounding and initiate the formation of biofilm at an alarming rate, which is much faster in diabetic patients [3]. Conventional antibiotics such as streptomycin and vancomycin combat bacterial infections by causing irreparable damage to the bacteria through various mechanisms like inhibition of essential proteins [4]. Antibiotics not only treat microbial infections but also play an indispensable role in preventing severe infections in chronically ill patients such as diabetes and renal disease or who have had complex surgeries [5, 6]. Emergence of AMR along with massive reports of overdose or misuse of administered antibiotics are responsible for impotency of existing antibiotics [7]. From the year of 2050, microbial infections will account for the death of more than 10 million people per year [8]. Moreover, multidrug resistance (MDR) bacteria can produce enzymes to degrade or inactivate antibiotics, or to alter bacterial efflux pumps, targeting binding sites, and entry ports to inhibit antibiotics [9]. With the deteriorating efficiency of currently available antibiotics and growing AMR, it is of great importance to discover alternative strategies against infectious diseases.

To circumvent the challenges associated with AMR, various carbon-, metal/metal oxide-, and two-dimensional (2D) nanomaterial-based antimicrobial modalities have been developed to disintegrate bacteria via complex mechanisms, including disruption of bacterial membranes, proteolysis, degradation of DNA, or elevated oxidative stress [10–13]. Owing to their small sizes, large surface area, and mechanical strength, these particles can strongly and directly contact with bacterium membranes to achieve desirable antimicrobial activities [14–16]. These nanomaterials that are often recognized as “endogenous antimicrobial” can delay bacterial damage, and thus repeated administration inevitably leads to AMR [17]. In addition, cytotoxicity of metal ions leached out from nanoparticle surfaces is also a concern [18]. Although surface passivation is feasible using small organic ligands, polymers, and biomacromolecules, antibacterial activity is compromised [19]. Therefore, development of biocompatible nanomaterials that offer multiple routes of biocidal action in vivo systems or clinical settings is a colossal challenge.

Ultrathin 2D nanomaterials with the lateral size larger than 100 nm and thickness of only a single- or few-atoms thick (< 5 nm) represent an emerging class of antimicrobials, mainly due to their huge specific surface and faster electron transfer ensuring sufficient surface-active sites [20–22]. For example, Zhao et al*.* reported highly catalytic reduced graphene oxide nanosheets for antimicrobial therapies [23]. The nanosheets provided therapeutic potency against MDR bacteria; however, they were non-responsive to near-infrared (NIR) irradiation, and thus they do not allow in-depth tissue penetration and generation of localized heat (> 50 °C) to inactivate the bacteria. Another report, Ding et al*.* showed the synthesis of CuS/graphitic carbon nitride (g-C3N4) heterojunctions for NIR-laser irradiation-assisted photothermal therapy [24]. Although the heterojunctions induced faster electron transfer to promote photocatalytic performance, they could not generate H_2_O_2_.

Currently, MXene is a fast-growing family of 2D materials, comprised of transition metal carbides, carbonitride, and nitrides with a general formula of M_n+1_X_n_T_x_, where M is an early transition metal (e.g., Sc, Ti, V, Cr, Zr, Nb, Mo, Hf), X is carbon and/or nitrogen, and T_x_ is a surface terminal group (–F, –OH, –O, etc.) [25–27]. Owing to the fascinating properties including hydrophilicity, biocompatibility, conductivity, and photothermal properties, MXene has shown potential for biomedical applications [28, 29]. Rasool and coworkers proposed that the antibacterial activity of colloidal Ti_3_C_2_T_x_ nanosheets was originated from direct contact assisted oxidative stress-induced membrane disruption [30]. Size-dependent antibacterial properties of MXene have been reported; smaller ones exhibited a more substantial antimicrobial effect due to their sharp edges [31]. Recently, Li et al*.* demonstrated the synergistic therapy of bismuth sulfide/titanium carbide MXene (Bi_2_S_3_/Ti_3_C_2_T_x_) heterojunctions against microbial pathogenesis, which resulted in rapid wound healing [32]. Nanocomposites extensively improved the photocatalytic generation of reactive oxygen species (ROS), due to formation of Schottky-type defects, which suppressed the recombination of NIR (808 nm)-induced electron–hole pairs. Work function-dependent interfacial engineering of Ti_3_C_2_T_x_ MXene significantly boosted the photocatalytic therapeutic action, but only against planktonic cells. In addition, most antimicrobial activities of MXene were shown in aqueous solutions with low ionic strength, mainly because of its poor stability in biologically complex fluids [33].

**Fig. 1:**
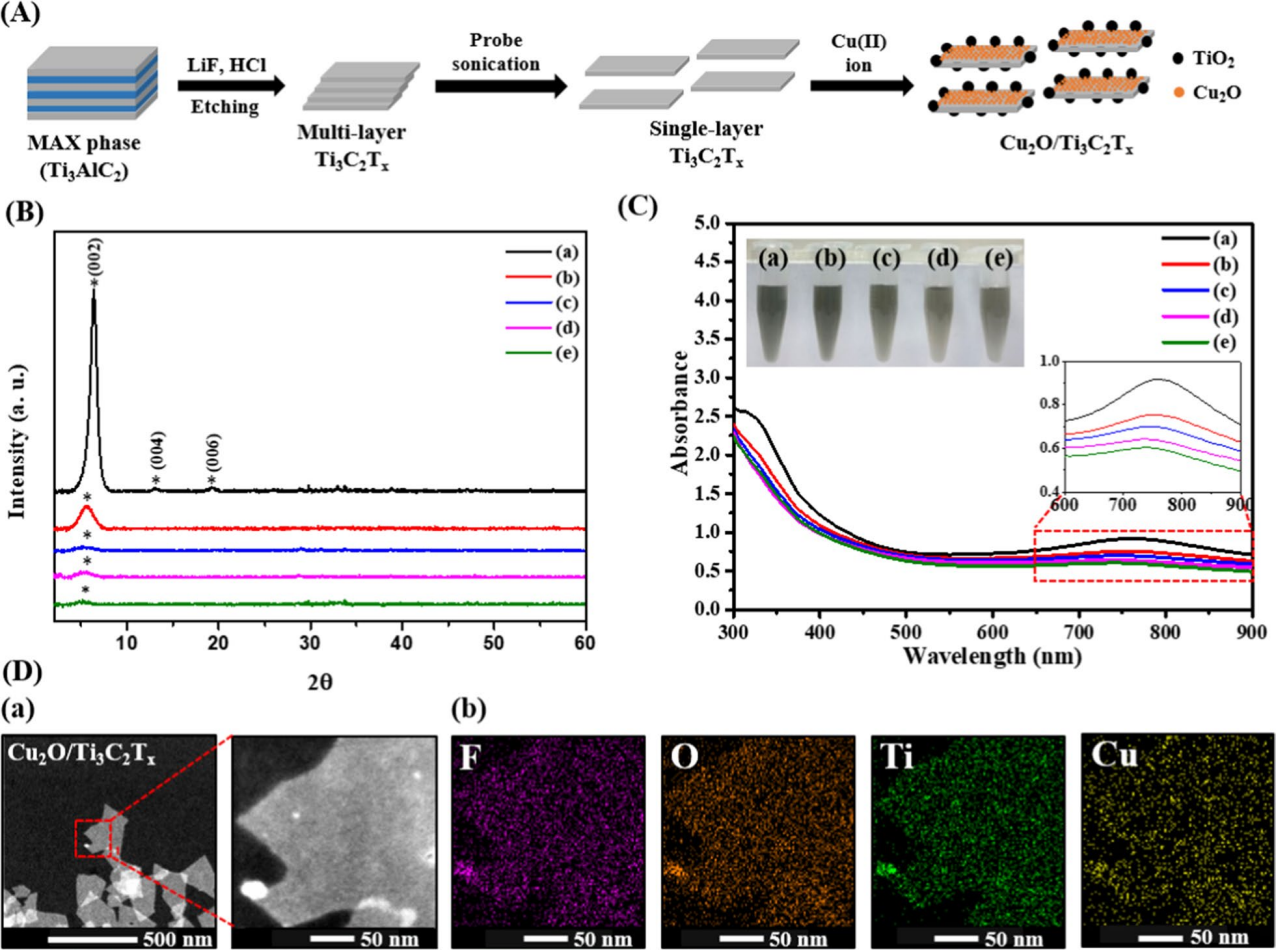
**A** Schematic representation of the synthesis process of Ti_3_C_2_T_x_—and Cu_2_O/Ti_3_C_2_T_x_ nanosheets. **B** XRD pattern and **C** UV–Vis absorption spectra of Ti_3_C_2_T_x_ nanosheets **a** before and **b**–**e** after incubation with **b** 10, **c** 25, **d** 50 or **e** 100 μg mL^−1^ of CuCl_2_ for 1 h. Asterisks in **B** indicate peaks that are assigned to Ti_3_C_2_T_x_ nanosheets. **D** **a** HAADF-STEM image of Cu_2_O/Ti_3_C_2_T_x_ nanosheets and corresponding **b** elemental mapping of F, O, Ti, and Cu

In this work, we prepared highly stable and aqueous dispersible single layer Ti_3_C_2_T_x_ MXene nanosheets via layer delamination and subsequent etching of the MAX precursor. After addition of Cu(II) ions, they were adsorbed onto the negatively charged and oxygenated surface of Ti_3_C_2_T_x_ MXene nanosheets and subsequently induced in situ formation of titanium dioxide (TiO_2_) and cuprous oxide (Cu_2_O) species through their redox reactions to yield Cu_2_O/Ti_3_C_2_T_x_ MXene nanosheets (heterostructures) (Fig. 1A). The Ti_3_C_2_T_x_ MXene nanosheets photogenerated electron–hole pairs upon NIR (650–850 nm) irradiation, meanwhile the surface Cu_2_O species boosted the production of superoxide (O_2_^•–^) radicals and subsequent transformation of them into hydrogen peroxide (H_2_O_2_). As-prepared Cu_2_O/Ti_3_C_2_T_x_ MXene nanosheets displayed broad-spectrum antimicrobial susceptibility, including MDR bacteria. Localized heating and cytotoxic reactive oxygen species are responsible for its superior antibacterial activity. In addition, the direct contact also caused irreparable membrane damage due to the sharp edges of nanosheets. The Cu_2_O/Ti_3_C_2_T_x_ Mxene nanosheets showed insignificant cytotoxicity and hemolytic activity against human skin cells (NIH-3T3 skin cells) and erythrocytes, respectively. The practicality of Cu_2_O/Ti_3_C_2_T_x_ Mxene nanosheets was demonstrated by curing diabetic mice with superficial wounds infected with methicillin-resistant *Staphylococcus aureus* (MRSA). Under photoirradiation, Cu_2_O/Ti_3_C_2_T_x_ MXene nanosheets eliminated microbial pathogenesis and promoted wound healing via angiogenesis, epithelialization, and collagen deposition.

## Results

### In situ formation of Cu_2_O on Ti_3_C_2_T_x_ MXene nanosheets

Rough surfaces of tightly stacked Ti_3_AlC_2_ flakes (i.e., commercially purchased MAX phase; Additional file 1: Fig. S1A) were etched with a freshly prepared mixture of hydrofluoric acid and lithium fluoride to remove aluminum to yield multilayer Ti_3_C_2_T_x_ nanosheets (i.e., MXene; Fig. S1B), which were then subjected to sonication to form well-dispersed single or few layers of Ti_3_C_2_T_x_ MXene nanosheets (Fig. S1C) [34]. Selected area electron diffraction (SAED) analysis revealed a distinct (002) plane of hexagonal Ti_3_C_2_T_x_ nanosheets (Additional file 1: Fig. S2A) [35]. The X-ray diffraction (XRD) pattern of Ti_3_C_2_T_x_ nanosheets exhibits a characteristic peak centered at 6.3º ascribing to (002) basal plane of MXene (Fig. 1B) [36]. The MXene nanosheets were further incubated with CuCl_2_ at an ambient temperature for 1 h to deposit Cu(II) ions onto Ti_3_C_2_T_x_ nanosheets (Fig. 1A). The XRD pattern (Fig. 1B) shows a gradual decrease in the signal intensity of (002) plane upon increasing Cu(II) concentration, indicating disruption of the crystal structure probably due to in situ self-oxidation of MXene to form Cu_2_O and TiO_2_ species [37]. The X-ray photoelectron spectroscopy (XPS) analysis of Ti_3_C_2_T_x_ MXene (17.2%/47.0%/21.2%) shows that relative abundance of Ti^+^/Ti^2+^/Ti^3+^ is significantly reduced after Cu deposition (Additional file 1: Fig. S3A), whereas Ti^4+^ (i.e., TiO_2_) is greatly increased [38]. The XPS spectra show characteristic peaks of Cu 2p_1/2_ and Cu 2p_3/2_ at 932.5 and 952.3 eV (Additional file 1: Fig. S3B), respectively, attributed to the presence of Cu_2_O species, supporting that the deposited Cu(II) ions reacted with oxygenated moieties on the surface of Ti_3_C_2_T_x_ MXene to transform into Cu_2_O to yield Cu_2_O/Ti_3_C_2_T_x_ nanosheets (heterostructures) through self-induced redox reactions [39]. No obvious characteristic diffraction peaks of as-formed TiO_2_ and Cu_2_O suggest that they are present on MXene in amorphous forms.

Transmission electron microscope (TEM), scanning electron microscope (SEM) and atomic force microscopy (AFM) show Cu_2_O/Ti_3_C_2_T_x_ nanosheets have an average size and thickness of ca. 200 and 1.84 nm, along with rough edges due to in situ formation of Cu_2_O and TiO_2_ species (Additional file 1: Figs. S4 and S5). Their UV–Visible absorption spectroscopy results are in good agreement with the XRD and XPS analysis, displaying the well-known characteristic wide range near-infrared (NIR) band (650–850 nm) of Ti_3_C_2_T_x_ MXene (Fig. 1C) apparently decreases upon increasing Cu(II) concentration mainly because of the self-oxidation induced structural disruption of Ti_3_C_2_T_x_ MXene (inset to Fig. 1C) [40]. The high-angle annular dark-field scanning transmission electron microscopy (HAADF-STEM; Fig. 1Da) coupled with energy-dispersive X-ray spectroscopy (EDX) elemental mapping indicates the coexistence of Ti, F, Cu, and O in Cu_2_O/Ti_3_C_2_T_x_ heterostructures and the bigger sized TiO_2_ NPs are formed on the edges of nanosheets (Fig. 1Db).

### Generation of O_2_^•−^/H_2_O_2_ by Cu_2_O/Ti_3_C_2_T_x_ heterostructures

**Fig. 2:**
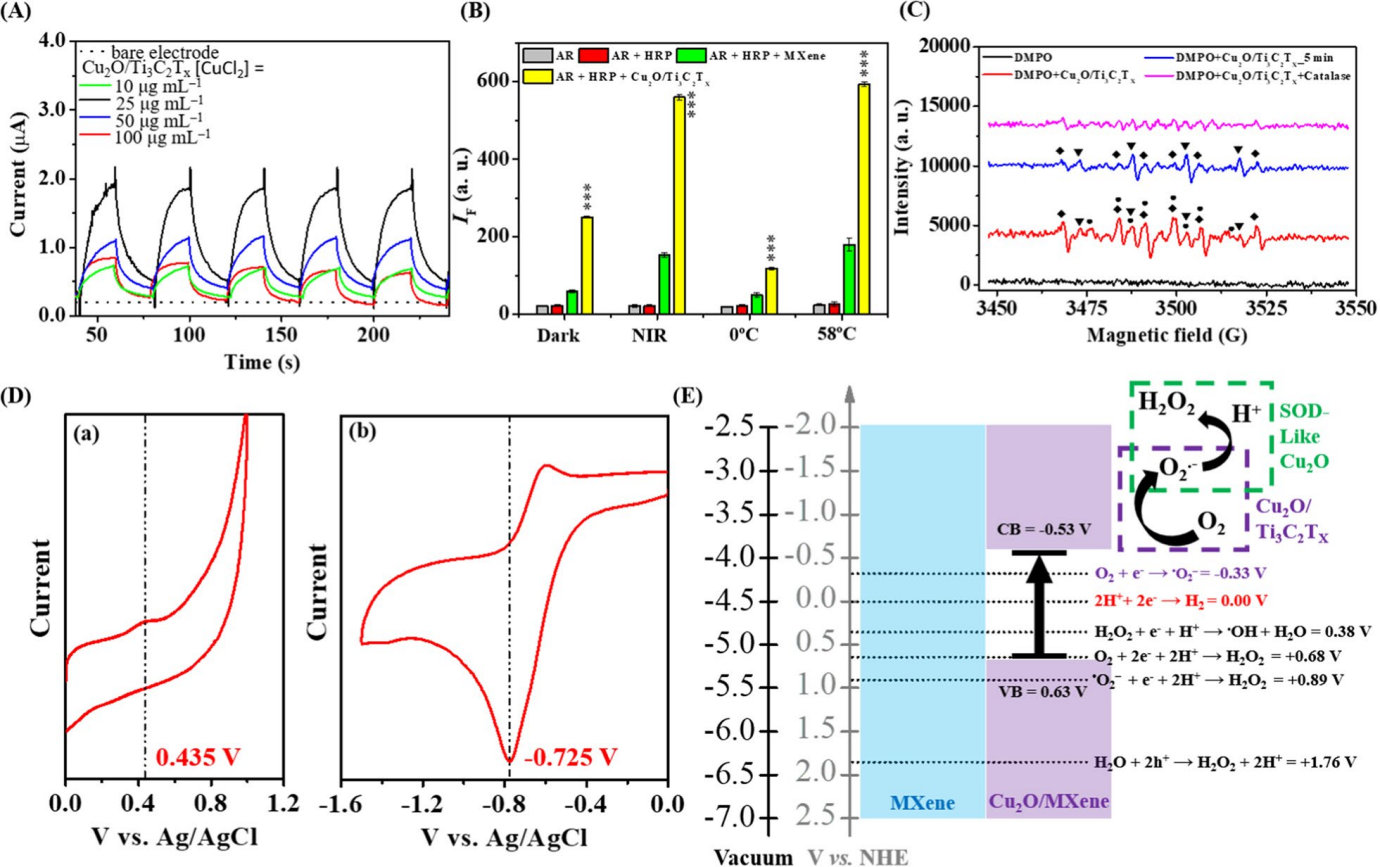
**A** NIR (808 nm, 0.53 W cm^−2^) separately induced photocurrent responses of Ti_3_C_2_T_x_ Mxene and Cu_2_O/Ti_3_C_2_T_x_ nanosheets in sodium sulfate solution (Na_2_SO_4_, 0.1 M) at − 0.25 V. Incubation of Ti_3_C_2_T_x_ Mxene with 10, 25, 50 or 100 μg mL^−1^ of CuCl_2_ for 1 h at room temperature to prepare Cu_2_O/Ti_3_C_2_T_x_ nanosheets. The ‘on’ and ‘off’ time interval in **A** is 20 s. **B** Fluorescence intensity of AR (50.0 μM) dispersed in PBS solution (pH 7.4) containing MXene or Cu_2_O/Ti_3_C_2_T_x_ nanosheets with HRP (0.1 unit mL^−1^) after incubation for 10 min. **C** ESR spectra of DMPO (10 mM) with Cu_2_O/MXene recorded immediately (red), and after 5 min (blue), and with the catalase (pink; 1.5 mg mL^−1^ equ. 12,000 U mL^−1^) in PBS solution, upon NIR irradiation (808 nm, 0.54 W cm^−2^, 10 min). The black rhombus (◆), triangle (▼), and dots (●) represent the signals of DMPO–⋅OOH (a_N_ = 1.42 G ${\mathrm{a}}_{\mathrm{H}}^{\upbeta }$ = 1.135 G), DMPO–⋅OH (a_N_ = a_H_^β^ = 14.9 G), and DMPO–H_3_C⋅ (*a*_N_ = 16.4 G, *a*^β^_H_ = 23.3 G), respectively. Concentration of Ti_3_C_2_T_x_ nanosheets or Cu_2_O/Ti_3_C_2_T_x_ nanosheets in **A**–**C** was fixed at 50 μg mL^−1^ (in terms of Ti_3_C_2_T_x_). **D** CV response of Cu_2_O/Ti_3_C_2_T_x_ nanosheets measured at **Da** positive and **Db** negative potential at a scan rate of 10 mV s^–1^ in the presence of an inert (i.e., ionic liquid) electrolyte solution. The Cu_2_O/Ti_3_C_2_T_x_ nanosheets prepared at 50 μg mL^−1^ of CuCl_2_ were used in **B**–**D**, Proposed energy band diagram of Cu_2_O/Ti_3_C_2_T_x_ nanosheets and possible reaction mechanisms for the enhanced production of ROS upon photoirradiation. The error bars in **B** represent the standard deviation of three repeated experiments. Asterisks in **B** indicate a statistically significant difference of the AR + HRP + Cu_2_O/Ti_3_C_2_T_x_ groups compared to those of AR + HRP + MXene groups (***p < 0.0001)

First, we demonstrated the capacity of Cu_2_O/Ti_3_C_2_T_x_ nanosheets to generate NIR-induced electron−hole pairs (Fig. 2A) [41]. Ti_3_C_2_T_x_ MXene could not generate photocurrent due to their intrinsic metallic/metal-like behavior upon NIR irradiation (808 nm, 0.53 W cm^−2^) [42]. On the other hand, Cu_2_O/Ti_3_C_2_T_x_ nanocomposites showed obvious photocurrent responses as compared to a bare electrode, mainly due to the formation of semiconducting Cu_2_O on the surface of nanosheets. The photocurrent (~ 1.83 × 10^−6^ A) produced by the Cu_2_O/Ti_3_C_2_T_x_ nanosheets ([Cu(II)] = 25 µg mL^−1^) is at least 2.4-fold higher than those (7.75 × 10^−7^ A) nanosheets prepared using 10 µg mL^−1^ of Cu(II), suggesting that amplified photocurrent response of Cu_2_O/Ti_3_C_2_T_x_ is attributed to the formation of heterostructures formed by the coupling of Cu_2_O with TiO_2_/Ti_3_C_2_T_x_ [43]. Further increase in Cu(II) concentration i.e., 50 or 100 µg mL^−1^ (in terms of Cu) resulted in a significant drop in the photocurrent, probably due to higher degree oxidation of Ti_3_C_2_T_x_ MXene (TiO_2_ > 94%, Additional file 1: Fig. S2). The presence of Cu_2_O in crystalline Ti_3_C_2_T_x_ nanosheets created an energy barrier (i.e., Schottky junction), which restricted the electron–hole pair recombination and facilitated the faster transfer of photogenerated electrons to the surface, leading to the enhanced photocurrent response [44]. Due to the substantial photoactivity through modulating electrons-holes, we further investigated the catalytic formation of hydrogen peroxide (H_2_O_2_) under NIR irradiation. Efficient generation of H_2_O_2_ was demonstrated through a combination of Amplex Red (AR) and horseradish peroxidase (HRP) assays [45]. HRP decomposes H_2_O_2_ to facilitate an electron transfer induced AR-oxidation to yield a highly fluorescent product resorufin (7-hydroxy-3H-phenoxazin-3-one; quantum yield: 0.83) [46]. As-prepared Cu_2_O/Ti_3_C_2_T_x_ heterostructures generated a comparable amount of H_2_O_2_ (10 μM), which is ~ fivefold higher than that of the Ti_3_C_2_T_x_ MXene at an ambient temperature under dark condition (Fig. 2B). Interestingly, the amount of H_2_O_2_ generated by Cu_2_O/Ti_3_C_2_T_x_ was further improved twofold when coupled with NIR irradiation (808 nm, 0.54 W cm^−2^, 10 min). In control experiments, reaction carried at 58 ºC (equivalent to the NIR induced temperature) with Cu_2_O/Ti_3_C_2_T_x_ nanosheets revealed a similar trend to that of NIR laser, indicating that spontaneous formation of H_2_O_2_ can be ascribed to the NIR induced in situ thermal gradients (Fig. 2B) [47].

The electron spin resonance (ESR) spectra of Cu_2_O/Ti_3_C_2_T_x_ heterostructures using 5,5-dimethyl-1pyrroline-N-oxide (DMPO) as a spin trap exhibited mixture of signals, corresponding to hydroxyl (⋅OH) and hydroperoxyl/superoxide (⋅OOH/O_2_^•–^) (Fig. 2C) [48]. In addition, a weak signal corresponding to methyl radical (H_3_C⋅) was also observed, ascribing to the copper-induced oxidation of DMPO. Upon incubation at an ambient temperature for 5 min, the peak for ⋅OH quartet (1:2:2:1) became more intense, and disappeared in the presence of catalase, suggesting the ⋅OH originated from H_2_O_2_ (Fig. 2C) [49]. Nevertheless, the ESR spectra show weak intensity sextet ascribing to ⋅OOH/O_2_^•–^ in the absence of NIR, under parallel conditions (Additional file 1: Fig. S6). Control group (only Cu(II)) did not reveal any obvious peak after incubating with DMPO in the absence or presence of NIR (data not shown). To confirm the formation of O_2_^•–^ as an intermediate for subsequent formation of H_2_O_2_, we employed a tetrazolium dye MTT [3-(4,5-dimethyl-thiazol-2-yl) 2,5-diphenyl tetrazolium bromide] reduction method to form it’s colored formazan, as reported previously [50]. Additional file 1: Fig. S7 indicates that Cu_2_O/Ti_3_C_2_T_x_ (Abs_576_ ~ 0.22) produced at least fourfold higher O_2_^•–^ as compared to the control (Abs_576_ ~ 0.05) without NIR irradiation, which is consistent with the low-intensity ESR peaks shown in Additional file 1: Fig. S6. In addition, spontaneous production of O_2_^•–^ was further improved threefold under NIR irradiation (Abs_576_ ~ 0.61), which is comparable to that provided by potassium dioxide (positive control; 0.5 mg mL^−1^).

2D structures with ultrafast charge carriers, huge surface area for redox reactions, broad-band absorption profile, and presence of heteroatom are the features of a modern photocatalyst [51]. Electrochemical behaviors of Cu_2_O/Ti_3_C_2_T_x_ heterostructures were studied by cyclic voltammetry (CV). The CV curves in a wide potential window were recorded to determine the position of valence band (VB; Fig. 2Da) and conduction band (CB; Fig. 2Db) [52, 53]. The energy band gap (E_g_) was calculated as 1.16 eV using Eq. (3):

1 $${\text{E}}_{{{\text{VB}}}} \left( {{\text{eV}}} \right) = - \left( {{\text{E}}_{{{\text{oxidation}}}} \,vs\,{\text{Ag/AgCl}} + 0.197 + 4.74} \right)$$

2 $${\text{E}}_{{{\text{CB}}}} \left( {{\text{eV}}} \right) = - \left( {{\text{E}}_{{{\text{reduction}}}} \,vs\,{\text{Ag/AgCl}} + 0.197 + 4.74} \right)$$

3 $${\text{E}}_{{\text{g}}} = {\text{E}}_{{{\text{CB}}}} - {\text{E}}_{{{\text{VB}}}}$$

As shown in Fig. 2E, Cu_2_O/Ti_3_C_2_T_x_ nanosheets can promote the thermodynamically favorable formation of O_2_^•–^ (O_2_ + e^−^ → O_2_^•–^ E^0^ =  − 0.33 V) upon NIR illumination, which were subsequently converted into H_2_O_2_ (O_2_^•–^ + e^−^ + 2H^+^  → H_2_O_2_ E^0^ =  + 0.89 V) by superoxide dismutase-like Cu_2_O species on the MXene surface [54]. Improved photoresponse of the Cu_2_O/Ti_3_C_2_T_x_ for the production of O_2_^•–^ is attributed to the formation of heterostructures that facilitated fast electron transfer and created catalytically active reaction centers with higher surface energy at the lattice heterojunctions between Cu_2_O and crystal facets of Ti_3_C_2_T_x_ in the nanosheets (Fig. 2E) [55]. Although the possibility of Fenton-like reaction of Cu(II)/Cu(I) for the generation of ⋅OH/H_2_O_2_ cannot be ruled out, we believe that the Cu_2_O/Ti_3_C_2_T_x_ heterostructures effectively inhibited the electron–hole pair recombination by creating an energy barrier, which promoted interfacial charge transfer for the generation of O_2_^•–^ [55].

### Photothermal effects of Cu_2_O/Ti_3_C_2_T_x_ heterostructures

**Fig. 3:**
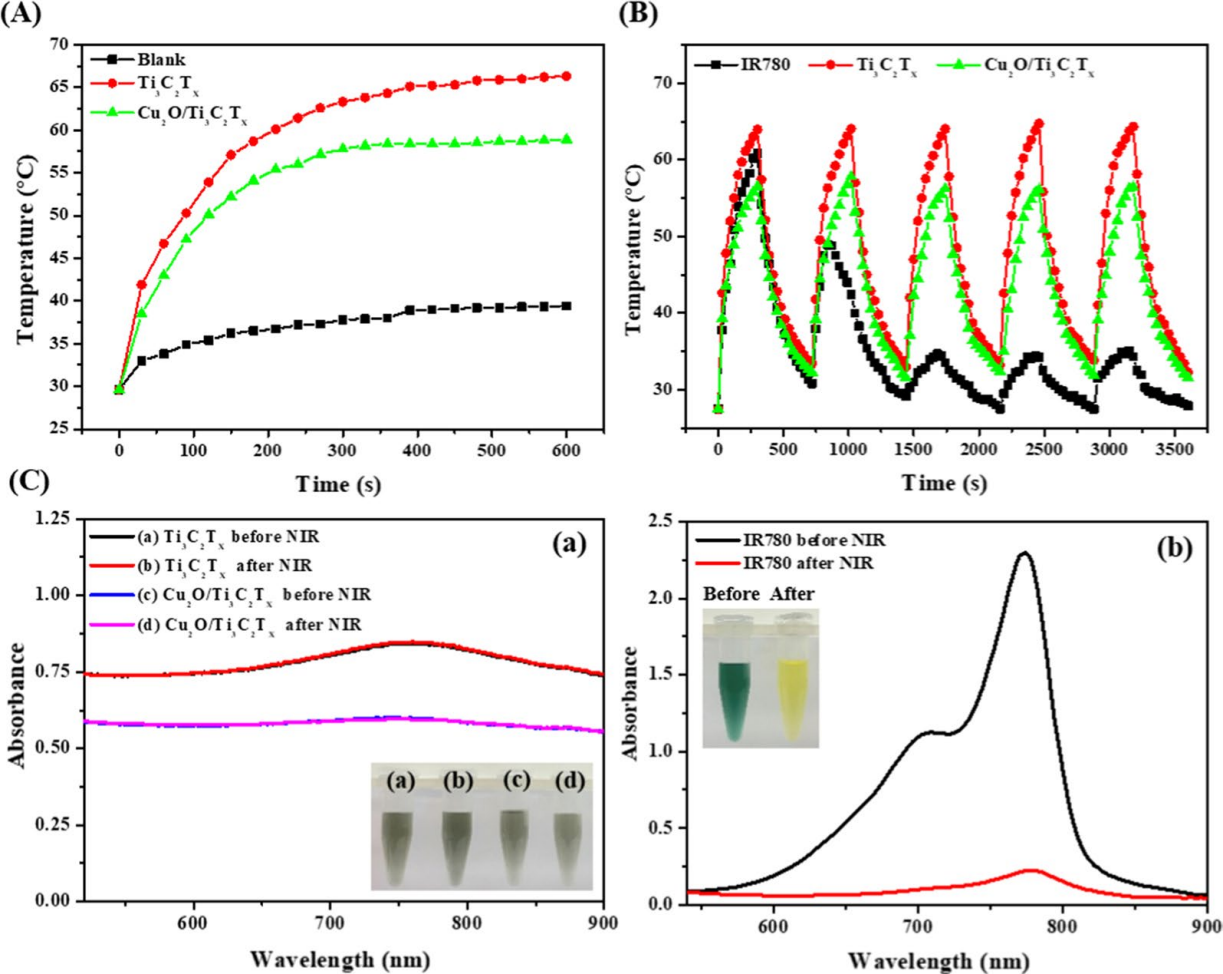
**A** Temperature profiles of Ti_3_C_2_T_x_ nanosheets and Cu_2_O/Ti_3_C_2_T_x_ nanosheets under NIR laser irradiation (808 nm, 0.54 W cm^−2^). **B** Thermal responses of Ti_3_C_2_T_x_ nanosheets, Cu_2_O/Ti_3_C_2_T_x_ nanosheets, and IR780 iodide dye (0.1 mM) dispersed in PBS solution under NIR laser irradiation for five consecutive switch on–off cycles. **C** UV–Vis absorption spectra of the corresponding solutions before and after five consecutive “on” and “off” cycles of NIR laser irradiation. Concentrations of Ti_3_C_2_T_x_ nanosheets and Cu_2_O/Ti_3_C_2_T_x_ nanosheets in **B** and **C** were both fixed at 50 μg mL^−1^ (in terms of Ti_3_C_2_T_x_). Inset to **C** shows the photographs of corresponding solutions

We further investigated the photothermal properties of MXene and Cu_2_O/MXene nanosheets. Temperature profiles of aqueous solution in the absence (control; PBS) and presence of 50 µg mL^−1^ (in terms of Ti_3_C_2__Tx_) of MXene or Cu_2_O/MXene nanosheets under continuous NIR laser irradiation (808 nm, 0.54 W cm^−2^) for 10 min are separately shown in Fig. 3A. As compared to PBS (~ 38 °C), both Ti_3_C_2_T_x_ MXene and Cu_2_O/MXene exhibited significant increases in temperature (> 57 °C) up to 300 s as a result of high light-harvesting ability and strong localized surface plasmon resonance (LSPR) effects [56]. The Ti_3_C_2_T_x_ nanosheets exhibited a characteristic SPR ascribed to the presence of the (002) crystal planes of Ti_3_C_2_T_x_, and the mobility of free charge carriers across the plane due to NIR irradiation that are indeed consistent with the natural specific frequency of plasmonic Ti_3_C_2_T_x_, as a result of the higher photothermal effects [56]. Both Ti_3_C_2_T_x_ MXene and Cu_2_O/MXene nanosheets showed obvious power density- and concentration-dependent temperature elevation profiles (Additional file 1: Fig. S8). The photothermal conversion efficiency (*η*) values of Ti_3_C_2_T_x_ and Cu_2_O/Ti_3_C_2_T_x_ nanosheets were determined by applying a slightly modified reported method to be ca. 53% and 49%, respectively (Additional file 1: Fig. S9) [57], which are comparable to or better than previous reports on MXene structures [58, 59]. Upon NIR laser irradiation, the oscillating electromagnetic field generated from the illuminated low-energy photons excites the free electrons of Ti_3_C_2_T_x_ nanosheets to coherently and collectively oscillate in their respective vibrational energy states [60, 61]. Then, the transitions within vibrational energy level allowed multiple collisions among the excited electrons, increasing the kinetic energy of each associated electron and then elevating the system temperature [62, 63]. In addition, relative to a commercially available photothermal agent (IR-780 dye; 0.1 mM), Ti_3_C_2_T_x_ and Cu_2_O/Ti_3_C_2_T_x_ displayed higher photothermal stability, as no significant decline in the temperature maxima was observed even after five consecutive cycles of on–off process (Fig. 3B). Absorption spectra showed no obvious color change in the Ti_3_C_2_T_x_ nanosheets and Cu_2_O/Ti_3_C_2_T_x_ nanosheets, while a significant photobleaching of IR-780 was observed under parallel conditions (Fig. 3C). The Cu_2_O/Ti_3_C_2_T_x_ nanosheets displayed considerable photo-to-heat conversion efficiency and photothermal stability, which makes them an excellent candidate for photothermal therapy applications.

### Potent antimicrobial activities

**Fig. 4:**
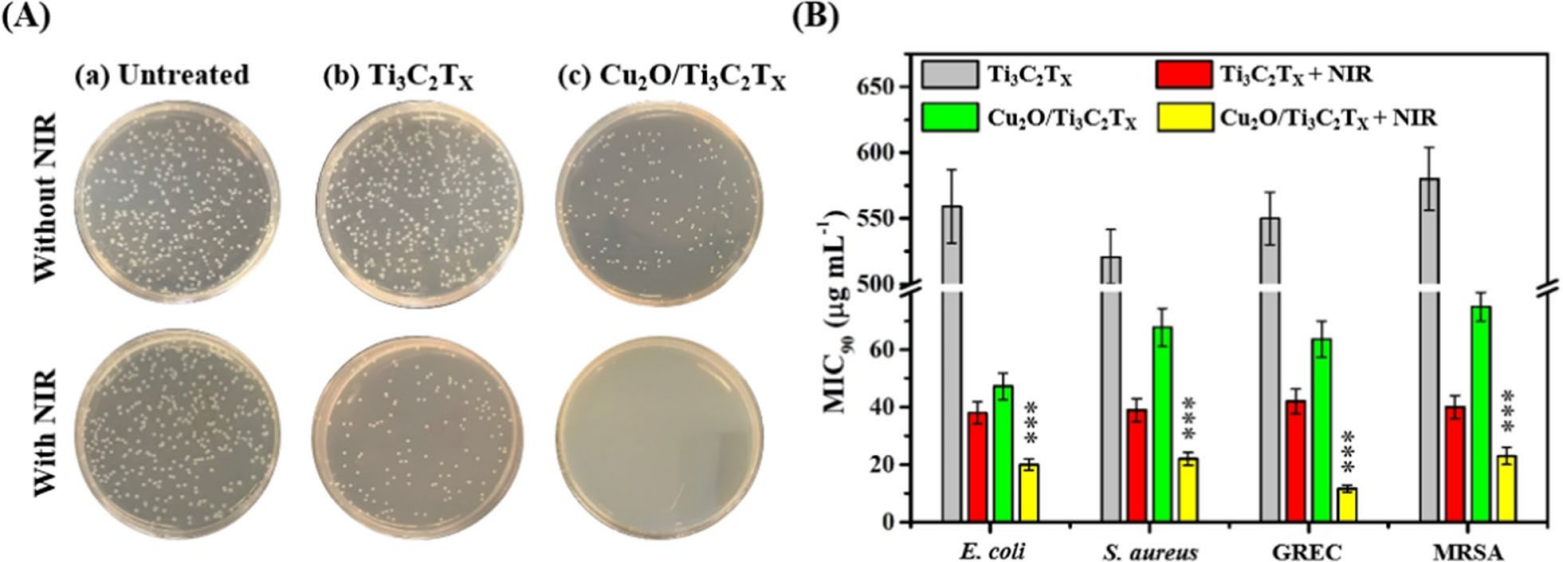
**A** Colony formation assays of *E. coli* on LB agar plates untreated and treated with 50 μg mL^−1^ (in terms of the Ti_3_C_2_T_x_) of Ti_3_C_2_T_x_ nanosheets or Cu_2_O/Ti_3_C_2_T_x_ nanosheets in PBS solution without and with NIR irradiation (808 nm, 0.54 W cm^−2^) for 10 min. **B** Comparative MIC_90_ values (in terms of Ti_3_C_2_T_x_) of Ti_3_C_2_T_x_ nanosheets and Cu_2_O/Ti_3_C_2_T_x_ nanosheets against four tested bacterial strains in the absence and presence of NIR laser (808 nm, 0.54 W cm^−2^, 10 min). The error bars in **B** represent the standard deviation of three repeated measurements. Asterisks in **B** show a statistically significant difference of the Cu_2_O/Ti_3_C_2_T_x_ + NIR groups compared to the pristine Ti_3_C_2_T_x_ groups (***p < 0.0001)

Owing to their excellent intrinsic catalytic activity and photothermal performance, we further evaluated the antibacterial properties of Cu_2_O/MXene nanosheets (with MXene as a control) against various bacterial strains, including one non-MDR gram-negative (*E. coli*), one MDR gram-negative (Gentamicin resistant *E. coli*; GREC), one non-MDR gram-positive (*S. aureus*), and one MDR gram-positive (MRSA) bacterial strains without and with NIR laser irradiation (808 nm, 0.54 W cm^−2^). Firstly, we demonstrated the multidrug-resistant nature of bacteria (i.e., MRSA) by examining their potency against several broad-spectrum antibiotics (Additional file 1: Fig. S10). Colony formation assay of *E. coli* untreated (230 ± 4 colony–forming units (CFUs); *n* = 3) and treated with Ti_3_C_2_T_x_ nanosheets and Cu_2_O/Ti_3_C_2_T_x_ nanosheets revealed 221 ± 5 and 115 ± 3 CFU (*n* = 3), respectively (Fig. 4A). The as-prepared Cu_2_O/Ti_3_C_2_T_x_ nanosheets showed negligible CFUs when coupled with the NIR laser irradiation (808 nm, 0.54 W cm^−2^, 10 min), while Ti_3_C_2_T_x_ nanosheets (137 ± 4 CFU, *n* = 3) exhibited much less inhibitory activity (Fig. 4A). The MIC_90_ (minimal inhibitory concentration required to inhibit > 90% of the bacterial population) values were determined by a standard serial dilution method (Fig. 4B) [64]. The MIC_90_ values for Cu_2_O/Ti_3_C_2_T_x_ nanosheets were determined to be > 47 µg mL^–1^ (in terms of Ti_3_C_2_T_x_), which were ⁓11-fold lower than that of Ti_3_C_2_T_x_ MXene (> 520 µg mL^–1^). As expected, under NIR irradiation MIC_90_ values of Cu_2_O/Ti_3_C_2_T_x_ were further reduced to ca. 11–23 µg mL^–1^ (in terms of Ti_3_C_2_T_x_). The Cu_2_O/Ti_3_C_2_T_x_ demonstrated remarkably superior antimicrobial performance upon NIR illumination due to the synergistic effect from catalytic and photothermal treatment.

**Fig. 5:**
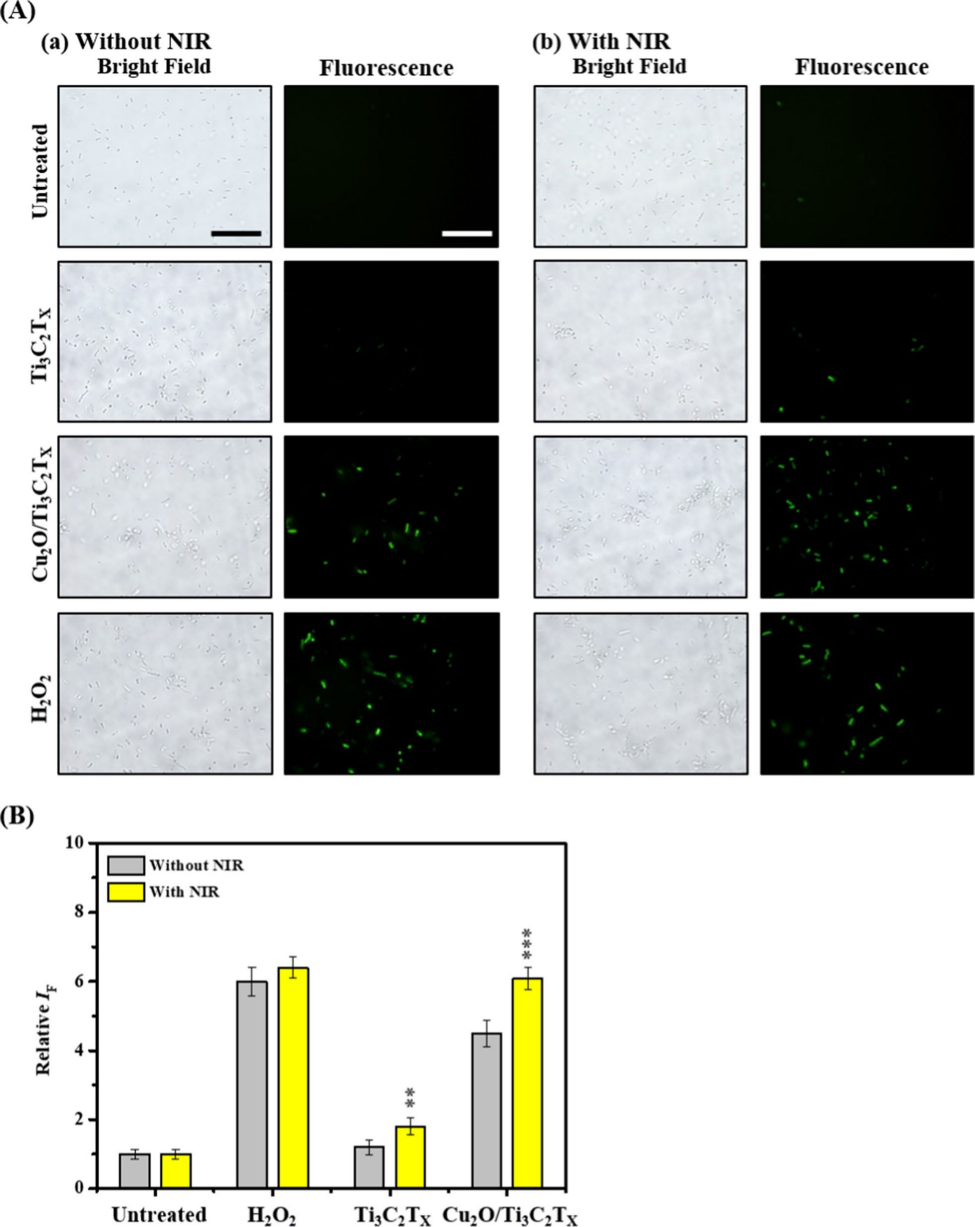
**A** The bright-field and fluorescence images of *E. coli* untreated and treated with 25 μg mL^−1^ (in terms of Ti_3_C_2_T_x_) of Ti_3_C_2_T_x_ nanosheets and Cu_2_O/Ti_3_C_2_T_x_ nanosheets, or H_2_O_2_ (20 µM) in PBS solution for 10 min without or with NIR laser irradiation (808 nm, 0.54 W cm^−2^). **B** Microscopic images were captured after the DCFH-DA staining. **B** Relative fluorescence intensity (*I*_F_) of DCF in *E. coli* suspensions was recorded at the excitation/emission wavelengths of 490/530 nm, respectively. The error bars in **B** represent the standard deviation of three repeated experiments. Scale bar in **A** is 10 μm. The error bars in **B** represent the standard deviation of three repeated experiments. Asterisks in **B** show statistically significant differences of the untreated, H_2_O_2_, Ti_3_C_2_T_x_, and Cu_2_O/Ti_3_C_2_T_x_ groups in the presence of NIR compared to those groups treated in the absence of NIR, respectively (**p < 0.001, and ***p < 0.0005)

We then studied intracellular ROS production capabilities of Ti_3_C_2_T_x_ and Cu_2_O/Ti_3_C_2_T_x_ nanosheets using the 2′,7′-dichlorodihydrofluorescein diacetate (DCFH-DA) assays [65]. We observed that *E. coli* treated with 25 µg mL^−1^ (in terms of Ti_3_C_2_T_x_ ) of Cu_2_O/Ti_3_C_2_T_x_ nanosheets under NIR laser irradiation induced remarkably higher ROS levels compared to those of Ti_3_C_2_T_x_ MXene or Cu_2_O/Ti_3_C_2_T_x_ nanosheets (without NIR irradiation) (Fig. 5). A small amount of ROS is generated in *E. coli* after being treated with Ti_3_C_2_T_x_ MXene with NIR laser irradiation, probably due to the photothermal effect of Ti_3_C_2_T_x_ MXene induced elevation of oxidative stress in bacteria. In addition, bacterium membrane integrity in the presence of MXene or Cu_2_O/Ti_3_C_2_T_x_ nanosheets without and with NIR irradiation were assessed using 3,3′-dietgyloxacarbicyanine (DiOC_2_) membrane potential staining assays (Additional file 1: Fig. S11) [66]. Suspensions of *E. coli* incubated with Cu_2_O/Ti_3_C_2_T_x_ nanosheets under NIR treatment showed significantly low red fluorescence intensity due to higher degree of depolarization of bacterial membranes, compared to the Ti_3_C_2_T_x_ MXene or Cu_2_O/Ti_3_C_2_T_x_ nanosheets without photoirradiation (Additional file 1: Fig. S11B). TEM images of *E. coli* after treatment with Cu_2_O/Ti_3_C_2_T_x_ nanosheets (25 µg mL^−1^) under NIR laser irradiation shows an apparently disrupted bacterial membrane, while an intact cell membrane with no obvious morphological changes was observed in untreated groups (Additional file 1: Fig. S12). The interaction of Cu_2_O/Ti_3_C_2_T_x_ nanosheets with the bacteria was further investigated by energy-dispersive X-ray spectroscopy (EDS) measurements of the portion of *E. coli* treated with the nanosheets, displaying a high-intensity signal of Cu in the surroundings (Additional file 1: Fig. S12). Superior antibacterial activity of Cu_2_O/Ti_3_C_2_T_x_ nanosheets coupled with NIR laser irradiation was achieved mainly due to a combined effect of catalytic and photothermal activities.

### Biocompatibility of Cu_2_O/Ti_3_C_2_T_x_ nanocomposites

We employed the trypan blue exclusion method for determining the cell numbers and Alamarblue assays to investigate the cytotoxicity of Cu_2_O/Ti_3_C_2_T_x_ nanosheets against NIH-3T3 cells (CRL-1658, fibroblast cells from Swiss albino mouse embryo tissue). Ti_3_C_2_T_x_ or Cu_2_O/Ti_3_C_2_T_x_ nanosheets exhibited insignificant cytotoxicity (< 5%) against the tested fibroblasts up to 50 μg mL^−1^ (in terms of Ti_3_C_2_T_x_e), even after NIR irradiation (808 nm, 0.54 W cm^−2^, 10 min), which is much higher than MIC_90_ values of the Cu_2_O/Ti_3_C_2_T_x_ nanosheets. (Additional file 1: Fig. S13). ICP-MS analysis revealed that only 10% Ti and 1% Ti/2.5% Cu were leached from the surfaces of Ti_3_C_2_T_x_ and Cu_2_O/Ti_3_C_2_T_x_ nanosheets in cell culture medium after incubated for 24 h, respectively. The modified nanosheets offered remarkable biocompatibility mainly due to their high stability, which prevented the release of metal ions in cell culture media (DMEM containing 10% FBS) in the duration of 24 h. In addition to satisfactory in vitro cytotoxicity performance, the hemolytic assay showed negligible hemolysis of erythrocytes up to 400 μg mL^−1^ (in terms of Ti_3_C_2_T_x_) (Additional file 1: Fig. S14).

### Effective wound healing using Cu2O/Ti_3_C_2_T_x_ nanosheets coupled with NIR irradiation

**Fig. 6:**
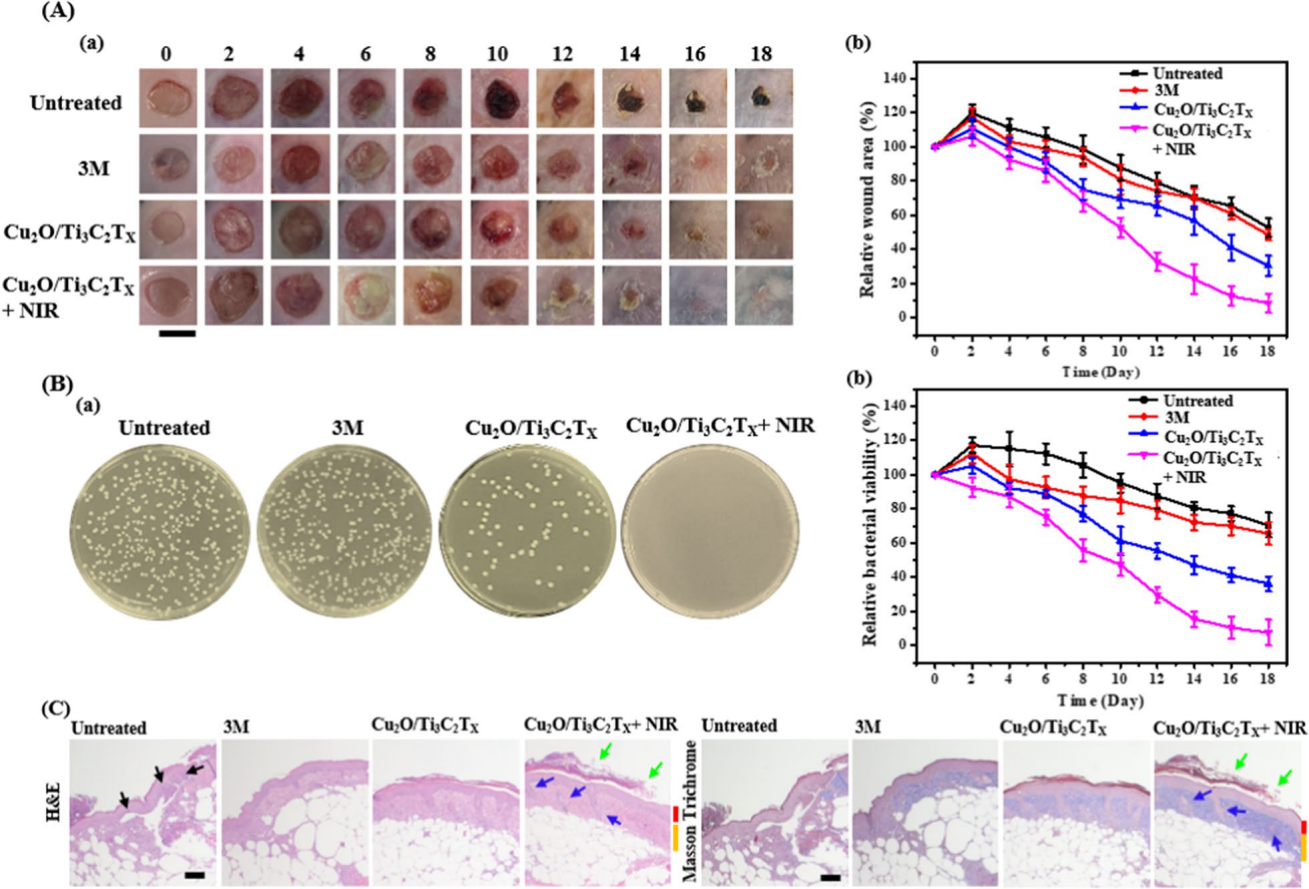
**Aa** Digital photographs and **b** relative wound closure area, **Ba** representative colony formation assays of MRSA collected from the wound site on day 14, and **b** bacterial viability of MRSA until day 18 from the wounds untreated and treated with 3 M bandage and 50 µg mL^−1^ (in terms of MXene) of Cu_2_O/Ti_3_C_2_T_x_ nanosheets without and with NIR irradiation (808 nm, 0.54 W cm^−2^, 10 min). The error bars **b** in **A** and **B** represent the standard deviation of three repeated measurements. **C** Histological analysis of tissue sections isolated on postoperative day 14 from the untreated and treated wounds. Red and yellow lines represent the thickness of the epidermis and dermis, respectively. Scale bar in **A** and **C** are 1 cm and 100 µm, respectively

Encouraged from aqueous stability, biocompatibility, and substantial antimicrobial performance in vitro, Cu_2_O/Ti_3_C_2_T_x_ nanosheets were applied for the treatment of infected wounds. *S. aureus* or their associated biofilms typically accounts for ca. 50% of nosocomial infection [67]. The optimal concentration of Cu_2_O/Ti_3_C_2_T_x_ nanosheets (50 µg mL^−1^; in terms of Ti_3_C_2_T_x_) was determined based on the results obtained from antibacterial assays (LB agar plates and MIC_99_ values) as shown in Fig. 4. In addition, biocompatible assays (Additional file 1: Figs. S13 and S14) also verified the optimal concentrations. The practicality of Cu_2_O/Ti_3_C_2_T_x_ nanosheets was validated with the treatment of surgical wounds infected with MRSA at the back of diabetic (db/db) mice (BKS.Cg- Dock7^m^ +/+ Lepr^db^/J, a model of Type 2 diabetes mellitus). Figure 6Aa shows time-course photographs of the wounds untreated (PBS solution) and treated with a commercial antibacterial bandage (i.e., 3 M; containing antimicrobial agent, benzalkonium chloride) or Cu_2_O/Ti_3_C_2_T_x_ nanosheets without and with NIR irradiation. Healthy mice took about 14 days to recover, while a similar-sized wound (10 mm) healed in at least 20 days or more in diabetic db/db mice [68]. Relative wound area on a postoperative day 12 of the Cu_2_O/Ti_3_C_2_T_x_ nanosheets-treated groups without and with NIR laser irradiation were reduced to ~ 57% and ~ 37%, respectively, ascribing to the effective reduction in bacterial colony count from the wound site when coupled with NIR irradiation (Fig. 6Ab). On the contrary, untreated wounds revealed negligible closure in the wound area and a high population of infected bacteria in the wound sites. Colony formation assay of MRSA collected on postoperative day 14 from wound sites co-treated with Cu_2_O/Ti_3_C_2_T_x_ nanosheets and NIR laser revealed insignificant bacteria growth (almost no colony formation) when compared to untreated ones (252 ± 14 CFU, *n* = 3) or 3 M treated (234 ± 17 CFU, *n* = 3) and to those treated with nanosheets without NIR irradiation (70 ± 5 CFU, *n* = 3) (Fig. 6Ba). The wound treated with Cu_2_O/Ti_3_C_2_T_x_ nanosheets under NIR irradiation displayed enhanced antimicrobial activity towards MRSA infected tissues mainly because of the localized photothermal effect (~ 50 °C; Additional file 1: Fig. S15) and high levels of ROS (i.e., O_2_^•−^ and H_2_O_2_) produced in situ (Fig. 6Bb). In addition, the contribution of sharp edges-induced physical disruption of bacterium membrane cannot be excluded. Although the local temperature of ~ 50 °C is relatively higher, the mice can tolerate it for a short period (10 min). The photothermal ablation process can be controlled by adjusting the drug concentration (Additional file 1: Fig. S8A) and power density (Additional file 1: Fig. S8B) of the laser. Minimal damage to the skin cells was observed mainly because dosage (50 µg mL^−1^) and power density (0.54 W cm^−2^) employed for wound treatment is optimal for a shorter duration (10 min).

Moreover, histological evaluation of hematoxylin and eosin (H&E)- and Masson’s trichrome-stained tissue specimens collected from the infectious site on postoperative day 14, showed that untreated groups displayed a large number of immune cells (black arrow) (Fig. 6C). In chronic wounds, compromised neutrophil and macrophage phagocytosis protract the regeneration of skin fibroblasts, while bacteria consume major amount of complementary proteins. Such processes occur during the inflammatory phase of wound healing, which delays the healing process [69, 70]. Furthermore, less pliable erythrocytes in protracted proliferative phase are unable to deliver oxygen to the wounded site for tissue metabolism and collagen synthesis [71]. Blue color in Masson’s trichrome staining represents collagen fibers, and the intensity is attributed to the collagen content in that particular tissue specimens (Fig. 6C). We observed more collagen fiber deposition in the recovered dermal layers, after treatment with Cu_2_O/Ti_3_C_2_T_x_ nanosheets coupled with NIR irradiation. Efficient antimicrobial response of the Cu_2_O/Ti_3_C_2_T_x_ nanosheets in the presence of NIR irradiation promoted angiogenesis, epithelialization, and collagen synthesis and formation of blood vessels and hair follicles (blue arrow). The development of new network of blood vessels allows an adequate supply of nutrients and oxygen, which stimulate the growth of granulation tissue. Moreover, we also observed higher degree of epithelialization (green arrow) in the Cu_2_O/Ti_3_C_2_T_x_ nanosheets treated wounds under NIR irradiation, compared to other groups, implying full recovery of wounds (Fig. 6C).

## Discussions

In today’s world with fast-developing therapeutic advances, it is vital to investigate new and improved alternative strategies against bacteria and viruses, especially after witnessing the COVID-19 havoc. But with progressing improvements in tackling these issues, the multidrug-resistant bacteria has started to adapt according to conditions resulting in degradation of existing antibiotics and alteration of bacteria efflux pumps posing a severe threat to sustaining human life. To address these issues, numerous research has been actively harnessing the potential of two-dimensional nanomaterials and various carbon-, metal/metal oxide-based structures [10–13]. These materials are often referred to as “endogenous antimicrobial” and damage bacteria through direct contact with bacterium membrane. However, their long-term uses may lead to AMR [17]. On the other hand, the cytotoxicity of metal/metal oxide due to leaching of metal ions cannot be overlooked. This situation lays the foundation for developing bio-compatible nanomaterials capable of in vivo action through multiple routes targeting colossal challenges. The enhanced specific surface area and fast electron transfer ensure sufficient surface-active sites, particularly ultrathin 2D nanomaterials which has also been highlighted in Zhao et al*.* work using r-GO nanosheets for antimicrobial therapies [20–23]. However, r-GO was inable to provide in-depth tissue penetration or localized thermal effect to inactivate bacteria. Owing to various structural properties, MXene comes to light as a potential candidate [28, 29]. An appreciable amount of work has been going on the heterojunctions and inducing defects in these structures, but the drawback lies in using the aqueous solution with low ionic strength because of poor stability in biologically complex fluids [33]. Our developed Cu_2_O/Ti_3_C_2_T_x_ nanosheets have several advantages such as facile synthesis Cu_2_O/Ti_3_C_2_T_x_ nanosheets conducted at room temperature, and as-prepared nanosheets exhibited remarkable biocompatibility mainly due to their high aqueous dispersibility and low toxic natures of Cu and Ti. The Cu_2_O/Ti_3_C_2_T_x_ nanosheets possess high photo-to-heat conversion efficiency and characteristic NIR absorbance, which allowed the development of a lower energy-based bacterial eradication from infected wounds. Thus they have a great scope as an antimicrobial modality for various biomedical applications.

## Conclusions

We demonstrated a self-redox reaction of CuCl_2_ and Ti_3_C_2_T_x_ MXene to form TiO_2_ and Cu_2_O species in situ on the surfaces of Ti_3_C_2_T_x_ nanosheets. Under NIR irradiation, the surface Cu_2_O species on Cu_2_O/Ti_3_C_2_T_x_ nanosheets boost the production of H_2_O_2_. In addition, temperature rise in response to low energy photons acted as a thermal gradient, which contributed to the production of H_2_O_2_. The MIC_90_ values of as-prepared Cu_2_O/Ti_3_C_2_T_x_ nanosheets under NIR irradiation against tested bacteria was > tenfold lower than pristine Ti_3_C_2_T_x_ MXene. Broad-spectrum antimicrobial susceptibility to various bacteria, including MDR bacteria, was achieved due to systematically generated therapeutic response, at first, sharp-edges of nanosheets physically attacked bacterium membrane (direct contact-mediated bacterial disintegration), then, NIR irradiation induced localized heat (~ 58 °C, photothermal therapy), and enhanced ROS production (catalytic therapy). The Cu_2_O/Ti_3_C_2_T_x_ nanosheets showed insignificant cytotoxicity and negligible hemolytic activity against human skin cells (NIH-3T3 skin cells) and RBCs, respectively. In vivo efficacy of Cu_2_O/Ti_3_C_2_T_x_ nanosheets was proven by treating the MRSA-infected surgical wounds created on diabetic mice under photoirradiation. Topically administered Cu_2_O/Ti_3_C_2_T_x_ nanosheets efficiently eliminated microbial pathogenesis caused by MRSA to accelerate wound healing via angiogenesis, epithelialization, and collagen deposition. Intrinsic radical scavenging capabilities of MXene may contribute to accelerated wound healing. Anti-oxidant properties of MXene and the role of Cu_2_O in enhanced stimulation of immune cells is currently under investigation. Our developed, Cu_2_O/Ti_3_C_2_T_x_ nanosheets possess superior biocompatibility, photothermal efficiency, and catalytic properties, which makes them an ideal candidate for the treatment of infectious diseases and wound healing applications.

## Methods

### Synthesis of MXene and Cu_2_O/Ti_3_C_2_T_x_ nanosheets

Nanosheets were prepared through a previously reported minimally intensive layer delamination (MILD) method, with minor modifications [34]. 40 mL of HCl (9 M) was added slowly into a PTFE bottle containing 4 g of LiF, which was then stirred for 30 min (600 r.p.m.) at ambient temperature. To which, 4 g of Ti_3_AlC_2_ MAX powder was slowly added over the course of 30 min. Then, the mixture was heated at 40 °C under constant stirring for 24 h and then subjected to centrifugation at a relative centrifugal force (RCF) of 3000*g* (10 min, 25 °C) to collect the etched multi-layered MXene in the pellet. The obtained residues were repeatedly washed with ultrapure water until the pH value reached to ~ 6.0. The delamination of purified multi-layered MXene was carried out by probe sonicator (Pulse 150 Ultrasonic Homogenizer, Thomas Scientific, Swedesboro, NJ, USA) in degassed water, under a flow of argon (Ar) gas for 4 h. During sonication, the surrounding temperature was maintained at 4 °C, while the frequency was set to 37 kHz, and the power of amplitude was 80%. The resulting solution was then centrifuged (RCF 3000×*g*, 5 min, 27 °C) to separate the delaminated flakes (dark green; monolayer Ti_3_C_2_T_x_ or MXene) from the unexfoliated sheets and larger particles. The concentration of purified MXene (in terms of mg mL^−1^) was determined using freeze-drying method. Freshly prepared monolayer MXene (1.0 mg mL^−1^, 0.5 mL) dispersed in sodium phosphate buffer (20 mM, pH 7.0) was separately incubated with CuCl_2_ solutions (20, 50, 100, 200 μg mL^−1^, in terms of Cu; 0.5 mL in water) for 1 h under continuous shaking (100 r.p.m.) at ambient temperature to obtain Cu_2_O/Ti_3_C_2_T_x_ nanosheets.

### Photoresponsive properties of Cu_2_O/Ti_3_C_2_T_x_ nanosheets

A continuous NIR laser (808 nm) with DPSSL driver II (Tangyu Precision Machinery Industry Co., Ltd., Taipei, Taiwan) was used to evaluate photocatalytic and photothermal performances of nanosheets. Temperature profiles of 50 µg mL^−1^ (in terms of Ti_3_C_2_T_x_) of as-prepared Ti_3_C_2_T_x_ MXene and Cu_2_O/Ti_3_C_2_T_x_ MXene dispersed in phosphate-buffered saline (PBS, pH 7.4, containing 137 mM NaCl, 2.7 mM KCl, 10 mM Na_2_HPO_4_, and 2.0 mM KH_2_PO_4_; 1 mL) solution under NIR laser irradiation (0.54 W cm^−2^) were continuously recorded using a TFC–305A Type K single input thermocouple thermometer (Yi Chun Electrics Co., Ltd., Taipei, Taiwan) for 10 min.

A freshly prepared Amplex Red (AR; 50 µM) was added into a 1.0 mL vial containing Ti_3_C_2_T_x_ or Cu_2_O/Ti_3_C_2_T_x_ nanosheets (50 µg mL^−1^; in terms of Ti_3_C_2_T_x_) dispersed in PBS solution with horseradish peroxidase (HRP; 0.1 unit mL^−1^) to estimate the catalytic generation of H_2_O_2_ [65]. The aliquots were then incubated at 0 °C, 58 °C, and at ambient temperature separately in dark and under NIR irradiation (808 nm, 0.54 W cm^−2^) for 10 min. Afterwards, the mixtures were subjected to measure the fluorescence intensity at an excitation/emission of 540/590 nm using a monochromatic microplate spectrophotometer (Synergy 4, Biotek Instruments, Winooski, VT, USA). We employed 5,5-dimethyl-1-pyrroline-*N*-oxide (DMPO) as a spin trap to perform Electron Spin Resonance (ESR) spectroscopy analysis for the detection of ROS. The ESR parameters are as follows: microwave power, 15 mW; microwave frequency, 9.8 gHz; scan range, 100 G; time constant, 0.328 s; sampling time, 20 ms; receiver gain, 30; modulation frequency, 100 kHz; g-factor, 2.00627; modulation amplitude, 0.1 mT.

### Determination of superoxide free radical

A freshly prepared MTT (1.0 mM) was added into a 1.0 mL vial containing Cu_2_O/Ti_3_C_2_T_x_ nanosheets (50 µg mL^−1^; in terms of Ti_3_C_2_T_x_) dispersed in PBS solution (pH 7.4) to assess the formation of MTT-formazan in the absence and presence of NIR irradiation (808 nm, 0.54 W cm^−2^, 10 min). MTT incubated with KO_2_ (0.5 mg mL^−1^) served as a positive control. Afterwards, as obtained purple-colored precipitates were dissolved in 50% isopropanol (1:1) and the absorbance at 576 nm was recorded immediately using a monochromatic microplate spectrophotometer (Synergy 4).

### Bacterial growth

*Staphylococcus aureus* (BCRC10781) and *E. coli* (BRBC 12438) were separately grown in Luria Bertani (LB) media, while MRSA (ATCC 43300) and GREC (BRCG 20703) were cultured individually in 1% penicillin and gentamicin containing LB broth, respectively. A single colony of each strain was plucked from solidified agar plates and inoculated in LB medium (1.0 mL). The cultures were then incubated at 37 °C under continuous shaking (200 r.p.m.), until the absorbance at 600 nm (OD_600_) reached to 1.0 (optical path length: 1.0 cm). Each of the bacterial suspension was centrifuged (RCF 3000×*g*, 10 min, 25 °C) and washed thrice with PBS solution. Finally, 100 μL of the *E. coli* suspension [5.0 × 10^3^ colony-forming unit (CFU) mL^−1^] was spread onto the solidified LB agar plates for the assessment of colony formation assay.

### In vitro antibacterial assays

The standard broth microdilution method determined the MIC_90_ (minimal inhibitory concentrations required to kill > 90% of total bacteria population) values against four different bacterial strains. Bacterial suspensions (1.0 × 10^4^ CFU mL^−1^) were incubated separately with CuCl_2_, Ti_3_C_2_T_x_, or Cu_2_O/Ti_3_C_2_T_x_ nanosheets in PBS solution (pH 7.4) at 37 °C under continuous shaking (250 r.p.m.) for 1 h and further incubated for 10 min at ambient temperature without and with NIR irradiation (808 nm, 0.54 Wcm^−2^, 10 min). Then, each of the diluted bacterial mixtures (100 μL) was spread onto the solidified LB agar plates, and CFUs were counted manually after 24 h incubation at 37 °C.

### Treatment of infected wounds using Cu_2_O/Ti_3_C_2_T_x_ nanosheets

The in vivo eradication of MRSA by Cu_2_O/Ti_3_C_2_T_x_ nanosheets was demonstrated using BKS Cg–Dock7^m^ +/+ Leprdb/J (db/db) male mice (5–6 weeks old, weighing 35–45 g). All animal experiments were conducted in accordance with the institutional guidelines of Care and Use of Laboratory Animals of National Taiwan University and granted by the Institutional Animal Care and Use Committee of the National Laboratory Animal Center, Taipei, Taiwan (IACUC Approval No. IACUC2012-037). The mice were anesthetized using a mixture of xylazine (117 mg kg^–1^) and Zoletil (5 mg kg^−1^). After shaving the dorsal skin of mice, the skin was disinfected using 70% ethyl alcohol (v/v) before aseptic surgery. An incision created circular wounds of ~ 10 mm in diameter on the skin of each mouse with a sterile stainless-surgical scissors. Microbial infection was induced by incubating the MRSA suspensions (1 × 10^8^ CFU mL^−1^; 50 µL) onto the wound site for 6 h. 14 days post-surgery, the wound sites were swabbed using a sterile cotton swab and cultured on LB agar plates for 24 h at 37 °C to confirm the MRSA infection. Then, Cu_2_O/MXene nanosheets were topically administered onto the wound site under NIR laser irradiation (808 nm, 0.54 W cm^−2^, 5 min; every other day). To evaluate the in vivo antimicrobial activity of as-prepared Cu_2_O/MXene nanosheets, tissue fluid (~ 10 µL) was swabbed from infectious wound site untreated and treated with Cu_2_O/Ti_3_C_2_T_x_ nanosheets without or with NIR laser irradiation on day 0, 2, 4, 6, 8, 10, 12, 14, 16, and 18 and separately grown on the LB agar plates to monitor the bacteria growth through colony formation assay. Photographs of the wound area were captured using a digital camera to observe the real-time progress of the infected wounds. Tissue sections containing the entire wound including surrounding healthy skin (dermis and subcutaneous tissue) were surgically removed on postoperative day 14 for histological evaluation. Collagen formation and skin histology of wounded tissue were studied by Masson’s trichrome and Hematoxylin and eosin (H&E) staining, respectively.

## Supplementary Information

**Additional file 1. **Additional methods, figures and tables.

## Data Availability

The data that support the findings of this study are available from the corresponding author upon reasonable request.
