# Supplementary material for: Self-redox reaction driven in situ formation of Cu2O/Ti3C2Tx nanosheets boost the photocatalytic eradication of multi-drug resistant bacteria from infected wound
Source: J Nanobiotechnology. 2022 May 19;20:235. doi: 10.1186/s12951-022-01428-3 (PMC9117998; doi:10.1186/s12951-022-01428-3)
Supplement: Supplementary file 1 — Additional file 1. Additional methods, figures and tables. [file 12951_2022_1428_MOESM1_ESM.docx]

Additional file 1

**Self-Redox Reaction Driven In Situ Formation of Cu_2_O/Ti_3_C_2_T_x_ Nanosheets Boost the Photocatalytic Eradication of Multi-Drug Resistant Bacteria from Infected Wound**

Ya-Ju Hsu^1,†^, Amit Nain^1,†^, Yu-Feng Lin^1^, Yu-Ting Tseng^1^, Yu-Jia Li^1^, Arumugam Sangili^1^, Pavitra Srivastava^2^, Hui-Ling Yu^1^, Yu-Fen Huang^3^, Chih-Ching Huang^4,5,*^ and Huan-Tsung Chang^1,^*

^1^Department of Chemistry, National Taiwan University, Taipei, 10617, Taiwan

^2^School of Basic Sciences, Indian Institute of Technology, Mandi (Kamand campus), Himachal Pradesh, 175005, India

^3^Institute of Analytical and Environmental Sciences, National Tsing Hua University, Hsinchu 30013, Taiwan

^4^Department of Bioscience and Biotechnology and Center of Excellence for the Oceans, National Taiwan Ocean University, Keelung, 202301, Taiwan

^5^School of Pharmacy, College of Pharmacy, Kaohsiung Medical University, Kaohsiung, 80708, Taiwan

*Corresponding authors.

E-mail addresses: huanging@ntou.edu.tw (C.-C. Huang), changht@ntu.edu.tw (H.-T. Chang)

^†^These authors contributed equally to this work

**Table of Contents**

Experimental Section…...…………………………………………………………………....…. S3

Figure S1. SEM images of MAX and MXenes………………………….……………………... S8

Figure S2. TEM and SAED pattern of Ti_3_C_2_T_x_ and Cu_2_O/Ti_3_C_2_T_x_ ...……..…….…………..… S9

Figure S3. XPS spectra of Ti_3_C_2_T_x_ and Cu_2_O/Ti_3_C_2_T_x_.....…………………………………….. S10

Figure S4. TEM and SEM of Ti_3_C_2_T_x_ and Cu_2_O/Ti_3_C_2_T_x_ …..………………………………... S11

Figure S5. AFM images of (A) Ti_3_C_2_T_x_ and (B) Cu_2_O/Ti_3_C_2_T_x_ nanosheets………………….. S12

Figure S6. ESR spectra of Cu_2_O/Ti_3_C_2_T_x_ ..…………….……………………………………... S13

Figure S7. MTT assays of Cu_2_O/Ti_3_C_2_T_x_ …..………….……………………………………... S14

Figure S8. Photothermal responses of Ti_3_C_2_T_x_ and Cu_2_O/Ti_3_C_2_T_x_ …..…………..…………... S15

Figure S9. The photothermal conversion efficiencies of Ti_3_C_2_T_x_ and Cu_2_O/Ti_3_C_2_T_x_ ..…….... S16

Figure S10. MIC_90_ values of antibiotics against *S. aureus* and MRSA …...……….….....….... S18

Figure S11. Membrane potential assays with *E. coli*………………..……..……..…….……... S19

Figure S12. TEM and SEM images of *E. coli*…………..…..…….…………………….…...... S20

Figure S13. *In vitro* cytotoxicity assays……………..…….…….………..……..……...…...... S21

Figure S14. Hemolytic activities of Cu_2_O/Ti_3_C_2_T_x_ …..………...….………………....….….... S22

Figure S15. Thermal profiles of the mice under NIR irradiation…..………………....….….... S23

**Additional experimental section**

**Materials**

Titanium aluminum carbide (Ti_3_AlC_2_, MAX, 400 mesh) was purchased from (11 Technology Co., Ltd. (Changchun, Jilin, China). Lithium fluoride (LiF, >99.99 %), sodium chloride, hydrochloric acid (37%), phosphoric acid (98%), sodium phosphate (96%), tris(hydroxymethyl)aminomethane (Tris), Luria Broth (LB), boric acid, and 2’-7’-dichlorodihydrofluorescein diacetate (DCFH-DA) were obtained from Sigma-Aldrich (St. Louis, MO, USA). Copper (II) chloride (CuCl_2_) and hydrogen peroxide (H_2_O_2_; 35%) were procured from Showa Chemical Industry Co., Ltd. (Tokyo, Japan). Amplex red was bought from Thermo Fisher (Eugene, OR, USA). Horseradish peroxidase (HRP) was purchased from Tokyo Chemical Industry (Tokyo, Japan).

**Characterization of Cu_2_O/Ti_3_C_2_T_x_ nanosheets**

Scanning electron microscopy (SEM) and transmission electron microscopy (TEM) images of the Ti_3_C_2_T_x_ and Cu_2_O/Ti_3_C_2_T_x_ nanosheets were captured using Hitachi S-4800 field-emission scanning electron microscope (Hitachi High-Technologies, Tokyo, Japan) and Philips/FEI Tecnai 20 G2 S-Twin transmission electron microscope (Hillsboro, Oregon, USA), respectively. Energy-dispersive X-ray spectrometer QUANTAX Annular XFlash® QUAD FQ5060 (Schaumburg, IL, USA) coupled with FE-SEM was employed to determine elemental composition of Ti, Cu, O, F in Cu_2_O/MXene *nanosheets*. For electron microscope, 10 μL of purified samples were dropped onto a carbon-coated copper grid and then dried at room temperature for 12 h before measurement. The X-ray diffraction (XRD) patterns were recorded by PANalytical X’ Pert PRO diffractometer (PANalytical B.V., Almelo, Netherlands) and Cu–Kα radiation (λ = 0.15418 nm). For XRD measurements, sample (150 μL) was coated onto Si substrate and dried overnight in a vacuum. X-ray photoelectron spectroscopy (XPS) analysis was performed using an ES-CALAB 250 spectrometer (VG Scientific, East Grinstead, UK) with Al Kα X-ray radiation as the X-ray source for photoejection. Binding energies were corrected using the C1s peak at 284.6 eV as an internal standard. The UV-visible absorption spectra of the Ti_3_C_2_T_x_ and Cu_2_O/Ti_3_C_2_T_x_ nanosheets were recorded using Evolution 220 UV-Visible spectrophotometer (Thermo Fisher Scientific Inc., Waltham, MA, USA).

**SEM and TEM Image of Bacteria**

*E. coli* suspensions (1.0 × 10^8^ CFU mL^−1^, 1.0 mL) were centrifuged (RCF 1000 *g*, 5 min, 25 °C) and washed three times with phosphate-buffered saline (PBS, pH 7.4, containing 137 mM NaCl, 2.7 mM KCl, 10 mM Na_2_HPO_4_, and 2.0 mM KH_2_PO_4_) solution. Bacterial cultures (1.0 × 10^8^ CFU mL^−1^) were then treated with Cu_2_O/Ti_3_C_2_T_x_ *nanosheets* ( 25 μg mL^−1^, in terms of Ti_3_C_2_T_x_) at 37 ºC for 1 h and further, incubated for 10 min in absence and presence of NIR laser irradiation (808 nm, 0.54 W cm^−2^). Each of the aliquots was centrifuged (1000 *g*, 5 min, 25 ºC) and washed thrice with PBS solution to remove unreacted Ti_3_C_2_T_x_ and were fixed using 4 % paraformaldehyde. For SEM, 10 µL (1.0 × 10^8^ CFU mL^−1^) was dropped onto Si-substrate and dried for 1h at room temperature, and images were captured using Hitachi S-4800 SEM (JEOL, Tokyo, Japan). 10 µL (1.0 × 10^8^ CFU mL^−1^) of each suspension was dropped onto the carbon-coated copper grid and incubated for 30 min at room temperature. After adsorption, the remaining solution was sucked out, and grids were dried at room temperature for 12 h.

**Reactive Oxygen Species (ROS) Assays**

Bacterial suspensions of *E. coli* (1.0 × 10^8^ CFU mL^−1^) were treated separately with 25 μg mL^–1^ (in terms of Ti_3_C_2_T_x_) of Ti_3_C_2_T_x_ or Cu_2_O/Ti_3_C_2_T_x_ nanosheets in PBS without and with NIR laser (808 nm, 0.54 W cm ^–2^) at ambient temperature for 10 min. The untreated and H_2_O_2_ (20 μM) treated *E. coli* culture used as the negative and positive controls, respectively. Each aliquot was centrifuged (RCF 1000 *g*, 10 min, 25 °C) and washed three times with PBS solution. Afterward, 2′,7′–dichlorodihydrofluorescein diacetate (DCFH–DA, 100 μM) was individually added to each bacterial mixture and further incubated for 30 min at ambient temperature. Then, 200 μL from each solution was transferred into a 96–well flat–bottom microplate to record the fluorescence intensities at an excitation/emission wavelengths of 490/530 nm, respectively, using a monochromatic microplate spectrophotometer. For microscopic images, 1.0 μL of the solution was dropped onto a glass slide and covered with a coverslip for bright field and fluorescence (Ex./Em. 460−480/510−530 nm) observation using an Olympus IX71 microscope (Tokyo, Japan).

**Bacterial Viability Assays**

*E. coli* cultures (1.0 × 10^8^ CFU mL^−1^) were and treated separately with 25 μg mL^–1^ (in terms of MXene) of Ti_3_C_2_T_x_ or Cu_2_O/Ti_3_C_2_T_x_ nanosheets in PBS solution for 1 h and further incubated for 10 min in the absence and presence of NIR laser irradiation (808 nm, 0.54 W cm^−2^) at ambient temperature. Untreated and 3-chlorophenylhydrazone (CCCP) treated groups served as the negative and positive control, respectively. Bacterial suspensions were centrifuged (RCF 1000 *g*, 10 min, 27 °C) and washed three times with PBS before fluorescence observation. A LIVE/DEAD BacLight Bacterial Viability Kit (Molecular Probes, Eugene, OR, USA) was employed to estimate live/dead bacteria ratio in each of the sample. Typically, a membrane potential indicator dye i.e., DiOC_2_ was added into each of the bacterial samples and incubated in the dark for 30 min at ambient temperature. Dye suspensions were then centrifuged (RCF 3500 *g*, 10 min, 27 °C) and washed thrice with PBS solution, to remove the unbound dyes. 1.0 µL from each aliquot was dropped onto the glass slide and covered with a coverslip for microscopic analysis. The green and red fluorescence were observed under excitation/emission filters of 460−490/≥525 nm (Olympus 1X71 microscope, Tokyo, Japan). Red and green fluorescence indicates the live and dead bacteria, respectively.

**Biocompatibility Evaluation Assays**

The cytotoxicity of Ti_3_C_2_T_x_ and Cu_2_O/Ti_3_C_2_T_x_ nanosheets was evaluated against NIH3T3 cells using Alamar blue assays (Thermo Fisher Scientific Inc.). Firstly, NIH3T3 cells (aneuploid immortal keratinocyte cell line from adult human skin, ATCC, Manassas, VA, USA) were cultured in Dulbecco’s Modified Eagle Medium (DMEM) supplemented with Fetal Bovine Serum (FBS; 10%), ampicillin (1%), L-glutamine (2.0 mM), and NEAA (1%) in a humidified incubator for 48 h at 37 °C. The cell number was determined by the trypan blue exclusion method. Then, NIH3T3 cells were seeded in 96-well plates (6 × 10^3^ cells/well) in DMEM for 12 h at 37 °C in a humidified incubator containing 5 % CO_2_. Various concentrations (0–50 μg mL^−1^; in terms of Ti_3_C_2_T_x_) of Ti_3_C_2_T_x_ or Cu_2_O/Ti_3_C_2_T_x_ nanosheets were treated separately with the cells for 24 h at 37 °C and further incubated at room temperature for 10 min without and with NIR laser irradiation (808 nm, 0.54 W cm^−2^). The nanomaterials were then replaced with a fresh growth medium and grown for another 24 h at 37 °C. Subsequently, the DMEM was removed, and cells were reacted with the Alamar blue solution (100 µL, 1x in DMEM containing 10% FBS) at 37 °C for 2 h. The fluorescence intensities at excitation/emission wavelengths of 550/600 nm were recorded for each well using a microplate reader.

**Hemolysis Assays**

A blood sample was withdrawn from the vein of a healthy Fischer rat (F344/NNarl, 180−200 g, 7−9 weeks). The red blood cell (RBCs) collection procedures were in compliance with the institutional guidelines and other concerned regulations. The blood sample was immediately centrifuged (3000 × g, 10 min, 4 °C) to separate the serum, and washed thrice with PBS solution (pH 7.4). Various concentration (0−400 μg mL^–1;^ in terms of Ti_3_C_2_T_x_) of Ti_3_C_2_T_x_ and Cu_2_O/Ti_3_C_2_T_x_ nanosheets were reacted separately with RBCs (~ 4 vol.%) dispersed in PBS (pH 7.4) for 1 h at ambient temperature. RBCs incubated with PBS and DI water were used as negative (0% hemolysis) and positive (100% hemolysis) controls, respectively. Each of the mixture was then centrifuged (RCF 3000 *g*, 10 min, 4°C), and absorbance at 576 nm (Abs_576_) of solutions in the supernatants were measured. The hemolysis (%) was determined using the following formula:

Hemolysis (%) = [(Abs_576 nanosheets_ – Abs_576 Blank_) / (Abs_576 Water_ – Abs_576 Blank_)


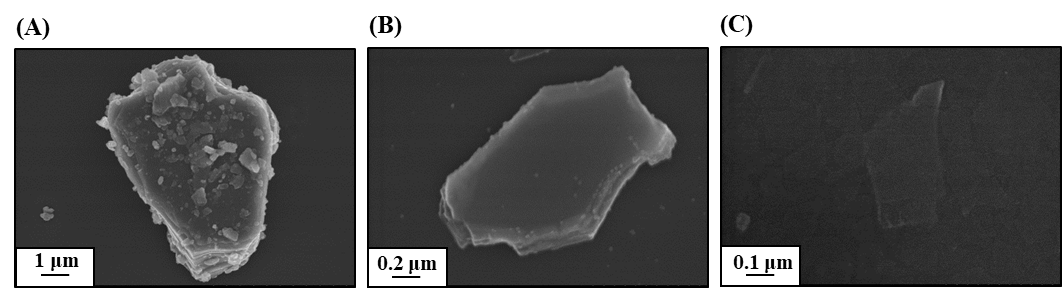


**Fig. S1.** SEM images of (A) Ti_3_AlC_2_ (MAX) phase, (B) multilayered Ti_3_C_2_T_x_, and (C) single-layer Ti_3_C_2_T_x_ nanosheets.


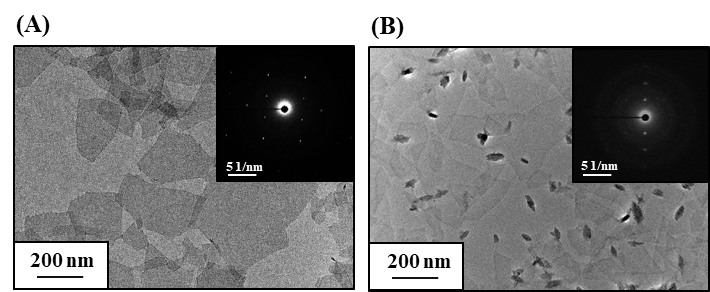


**Fig. S2.** TEM images of (A) Ti_3_C_2_T_x_ and (B) Cu_2_O/Ti_3_C_2_T_x_. Inset shows the corresponding SAED pattern.


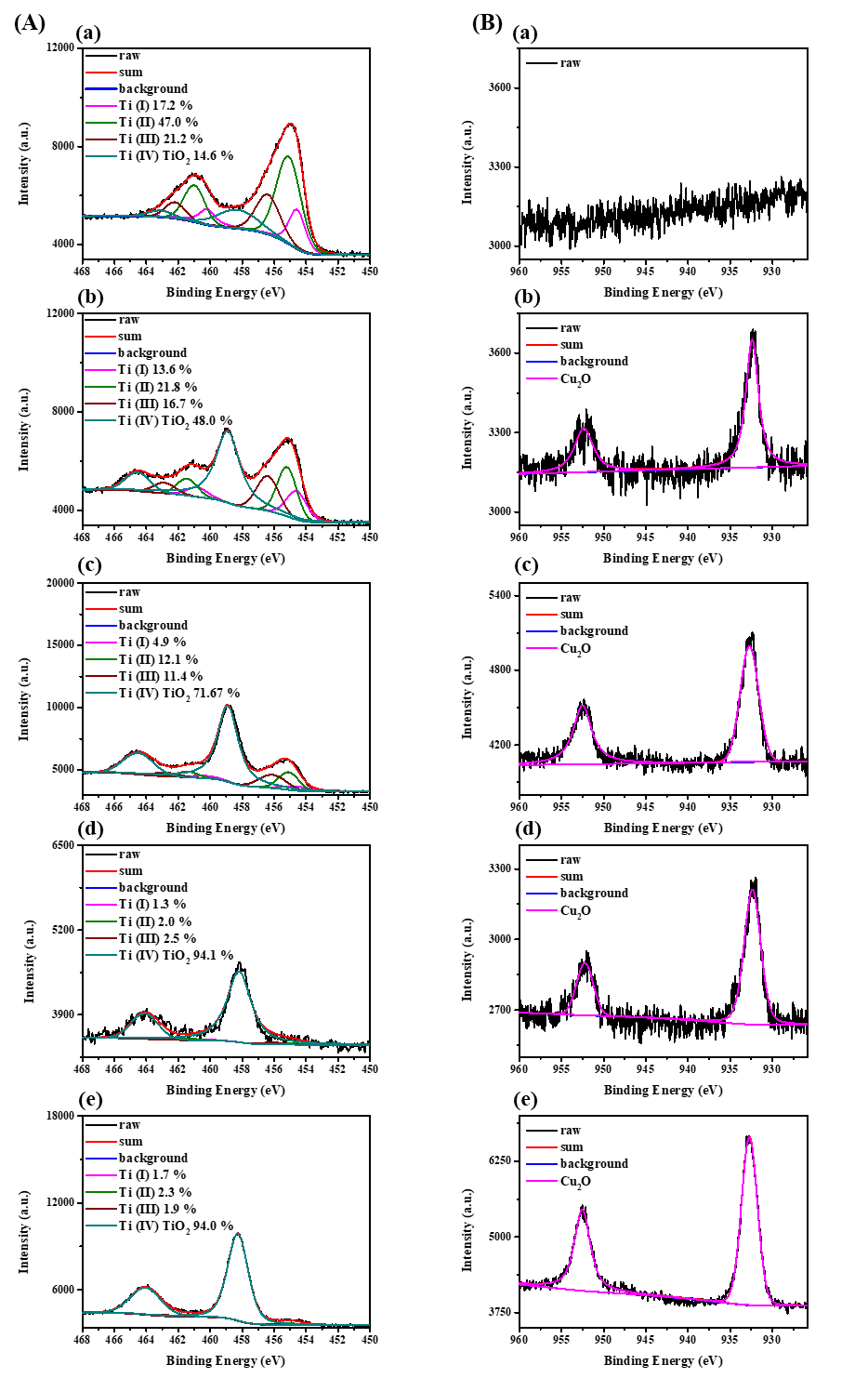


**Fig. S3.** Deconvoluted XPS spectra of (A) Ti 2p and (B) Cu 2p in Ti_3_C_2_T_x_ nanosheets (a) before and after being reacted with (b) 10, (c) 25, (d) 50 and (e) 100 μg mL^−1^ of Cu(II) ions


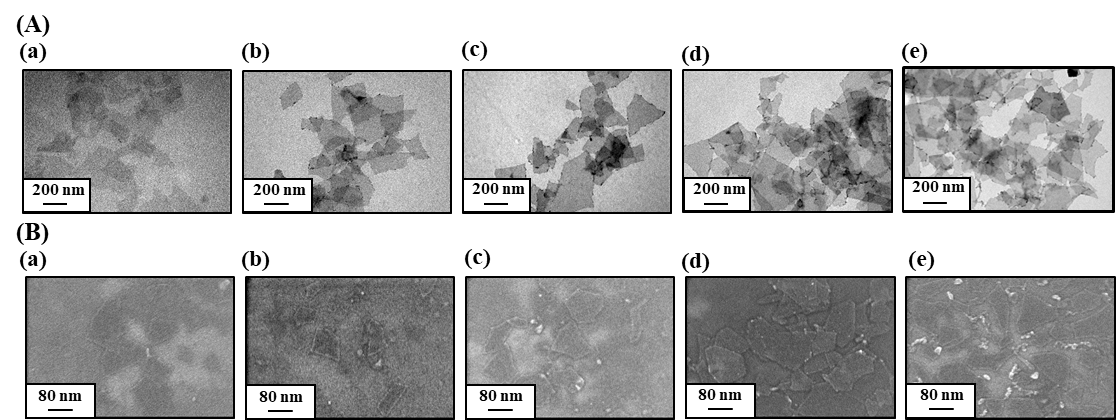


**Fig. S4.** (A) TEM and (B) SEM images of (a) Ti_3_C_2_T_x_ nanosheets and after reacted with (b) 10, (c) 25, (d) 50, and (e) 100 μg mL^−1^ of Cu(II) ions.


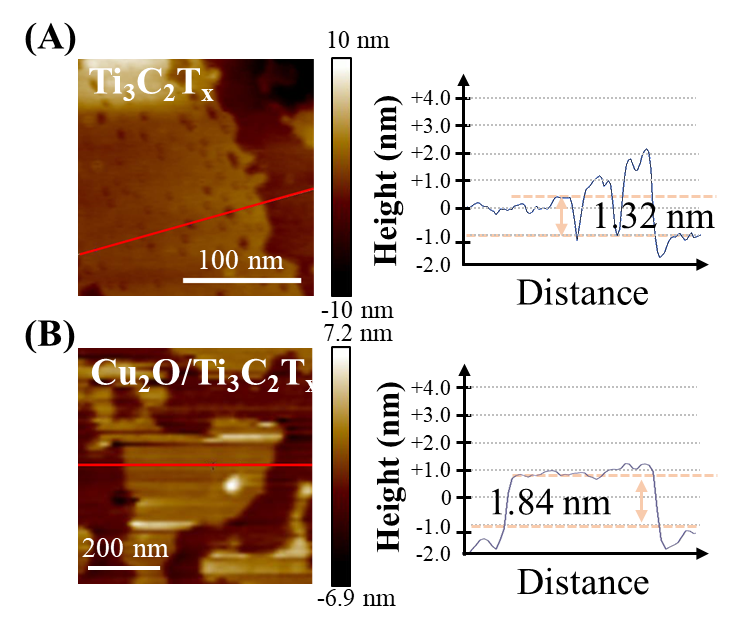


**Fig. S5.** AFM images of (A) Ti_3_C_2_T_x_ and (B) Cu_2_O/Ti_3_C_2_T_x_ nanosheets.


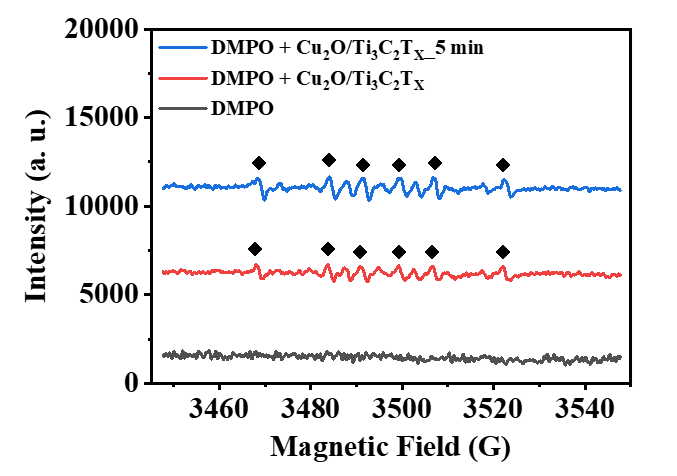


**Fig. S6.** ESR spectra of DMPO (10 mM) with Cu_2_O/Ti_3_C_2_T_x_ nanosheets recorded immediately (red) and after 5 min (blue) in PBS solution, without NIR irradiation. The black rhombus (◆) represents the signals of DMPO–^•^OOH (a_N_ = 1.42 G $a_{H}^{\beta}$ = 1.135 G). Other conditions were kept same as mentioned in Figure 2C.


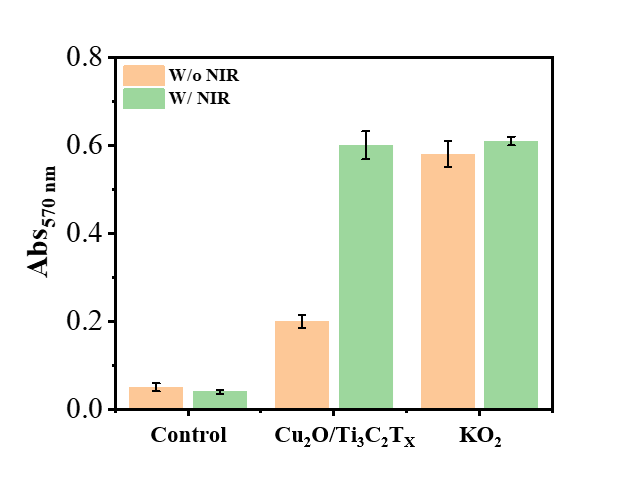


**Fig. S7.** Absorbance of MTT (1.0 mM) dispersed in PBS solution (pH 7.4) measured immediately before (control) and after separately incubating with Cu_2_O/Ti_3_C_2_T_x_ nanosheets (50 μg mL^−1^; in terms of Ti_3_C_2_T_x_) and KO_2_ (positive control; 0.5 mg mL^-1^) without and with NIR irradiation (808 nm, 0.54 W cm^−2^).


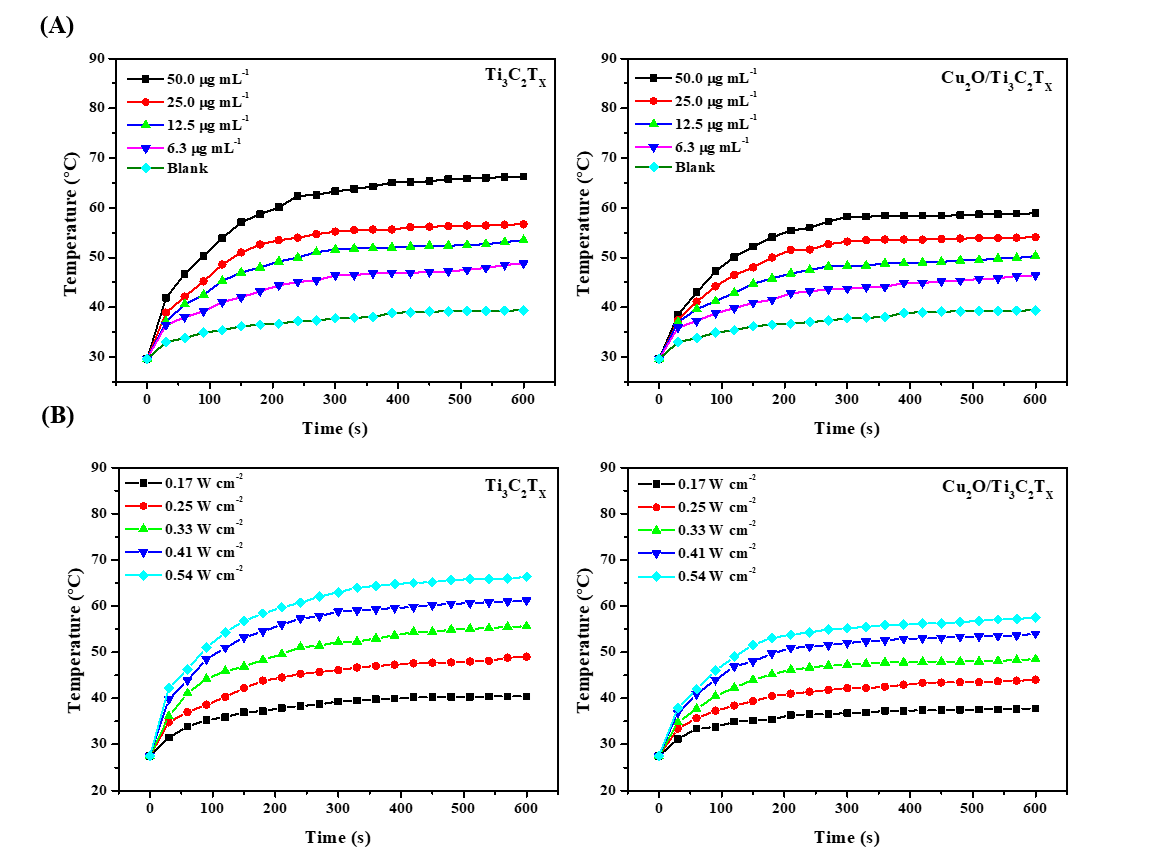


**Fig. S8.** (A) Concentration- and (B) power density-dependent temperature profiles of Ti_3_C_2_T_x_ and Cu_2_O/Ti_3_C_2_T_x_ nanosheets dispersed in PBS solution (pH 7.4). The power density of the 808 nm NIR laser in (A) was fixed at 0.54 W cm^−2^, and the concentrations of Ti_3_C_2_T_x_ and Cu_2_O/Ti_3_C_2_T_x_ nanosheets in (B) are both 50 μg mL^−1^ (in terms of Ti_3_C_2_T_x_).


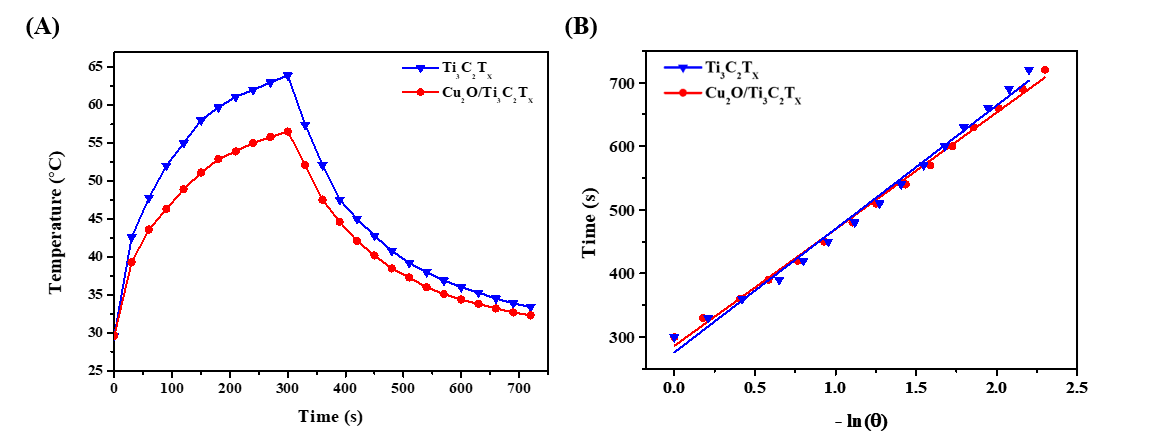


**Fig. S9.** (A) Temperature profiles of 50 µg mL^−1^ (in terms of Ti_3_C_2_T_x_) of Ti_3_C_2_T_x_ and Cu_2_O/Ti_3_C_2_T_x_ nanosheets in PBS solution (pH 7.4) under NIR laser irradiation (808 nm, 0.54 W cm^−2^) for 300 s and when the laser was turned off. (B) Linear plot time (*t*) from the cooling period *vs.* negative natural logarithm of dimensionless temperature constant (*θ*) (–ln θ *vs.* *t*) for the determination of time constant (*t*_s_).

Upon continuous NIR laser irradiation (0.54 W cm^−2^), the temperature changes of Ti_3_C_2_T_x_ and Cu_2_O/Ti_3_C_2_T_x_ nanosheets dispersed in PBS solution were recorded as a function of time. The laser was turned off once the system temperature reached its maxima, and then, heat transfer between the aqueous dispersion and buffer environment was calculated by closely monitoring the cooling temperature.

The photothermal conversion efficiency (*η*) of Ti_3_C_2_T_x_ and Cu_2_O/Ti_3_C_2_T_x_ nanosheets was calculated using the following equation:

$\eta=\frac{hS (T_{max}-T_{surr})-Q_{dis}}{I (1-{10}^{-\mathrm{Abs}_{808}})}$ (1)

where *h* is the heat transfer coefficient, *S* is the surface area of the sample holder, T*_max_* is the maximum temperature achieved by the system or equilibrium temperature, T*_surr_* is the ambient temperature, Q*_dis_* depicts the dissipated heat by the system, which was determined by irradiating the background buffer solution instead of nanosheets, and *I* is the fixed power density of the NIR laser. Abs*_808_* is the absorbance of Ti_3_C_2_T_x_ and Cu_2_O/Ti_3_C_2_T_x_ nanoshhets at 808 nm wavelength. For determining hS, first θ a dimensionless temperature constant was introduced into the system using T*_max_* and a new sample system time constant (*t*_s_):

$\theta=\frac{T-T_{surr}}{T_{max}-T_{surr}}$ (2)

and, $t_{s}=\frac{mC}{hS}$ (3)

where *m* is the total mass of the system excluding solvent (0.5 g) and *C* is the heat capacity of DI water (4.18 J g^−1^ °C^−1^). After the cooling stage, therefore, the time constant (*t*_s_) for heat transfer is determined by plotting the linear time data from the cooling period (after 500 s) *vs.* the negative natural logarithm of the dimensionless temperature constant (*θ*) (–ln*θ* *vs.* *t*) (**Fig. S8B**).

$t=t_{s}ln\theta$ (4)

Thus, according to Equation (3), *hS* of Ti_3_C_2_T_x_ and Cu_2_O/Ti_3_C_2_T_x_ nanosheets are estimated to be 0.002163 W °C^−1^ and 0.002289. *I* and Abs*_808_* were calculated to be 0.54 W cm^−2^, 0.88 of Ti_3_C_2_T_x_ and 0.66 of Cu_2_O/Ti_3_C_2_T_x_ respectively. Therefore, substituting the values of each constant into Equation (1), the heat conversion efficiency (*η*) of Ti_3_C_2_T_x_ and Cu_2_O/Ti_3_C_2_T_x_ nanosheets using an 808 nm laser was deduced to be 52.84 ± 6.2% and 48.62 ± 5.7% (*n* = 3), respectively.

**
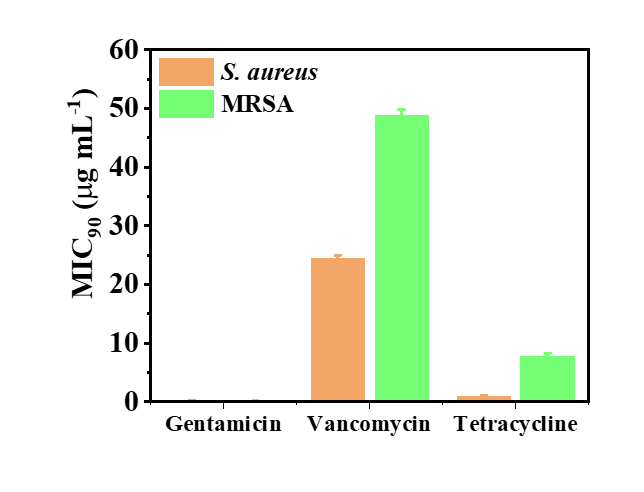
**

**Fig. S10** Comparative MIC_90_ values of gentamicin, vancomycin, tetracycline against non-resistant *S. aureus* and MRSA. Error bars represent the standard deviation of three repeated measurements.

**
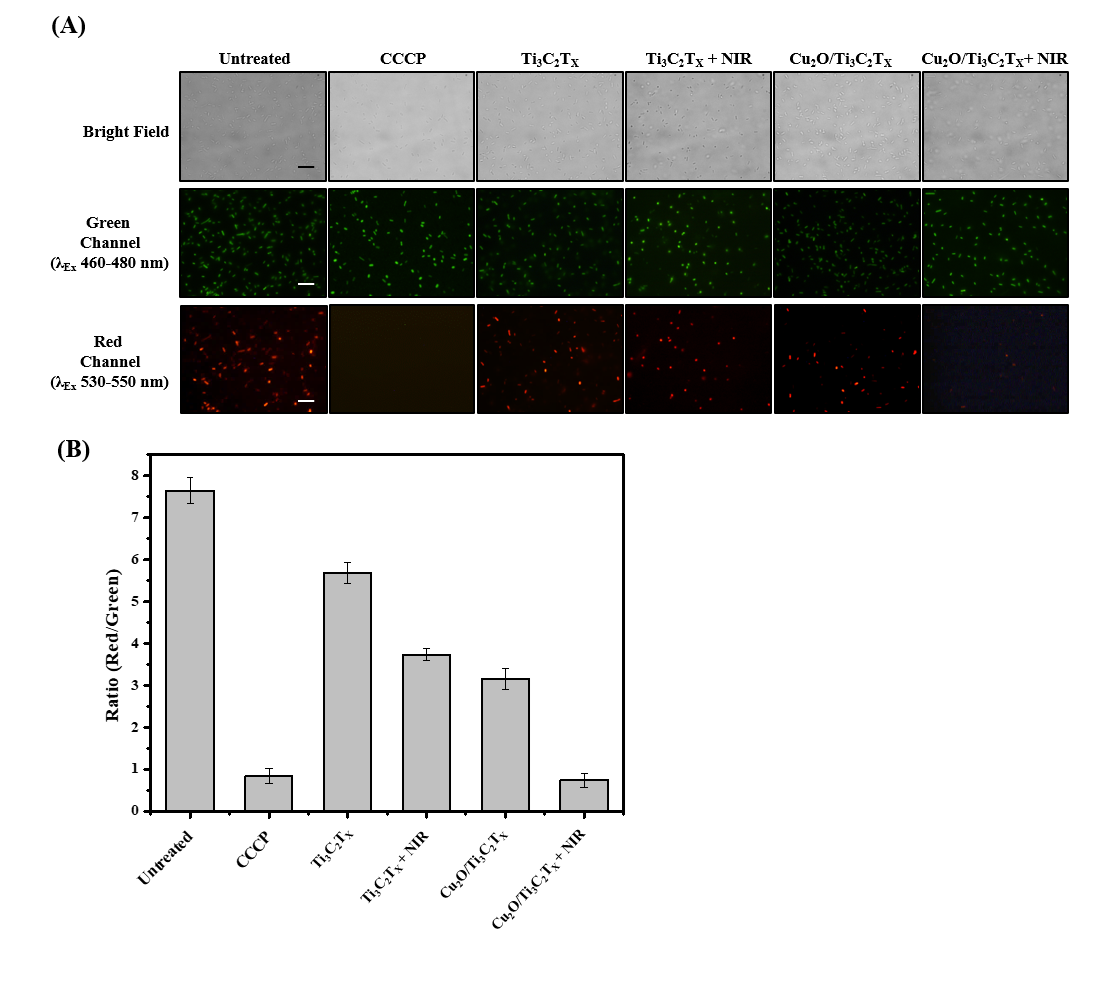
**

**Fig. S11.** (A) Bright-field and fluorescence images and Red/Green ratio plots obtained from DiOC_2_ staining of *E. coli* (10^8^ CFU mL^−1^) untreated and treated with 25 μg mL^-1^ (in terms of MXene) of Ti_3_C_2_T_x_, Cu_2_O/Ti_3_C_2_T_x_ nanosheets, or carbonyl cyanide 3-chlorophenylhydrazone (CCCP, 30 µM; positive control) without and with NIR irradiation (808 nm, 0.54 W cm^-2^) for 10 min in PBS solution. Scale bar is 10 μm.

The membrane potential change of the bacteria is readily detected and analyzed by using a fluorescent membrane-potential indicator dye, DiOC_2_. The *E. coli* exhibited green fluorescence at low concentrations of DiOC_2_, whereas DiOC_2_ accumulated in a healthy membrane is accompanied by a shift from green to red emission due to dye stacking. The ratiometric parameter (Red/Green fluorescence ratio) allows for measuring membrane potential in bacteria. After the treatment of Cu_2_O/Ti_3_C_2_T_x nanosheet_, the Red/Green ratio of the bacteria highly decreased as a result of depolarized bacterial membranes.


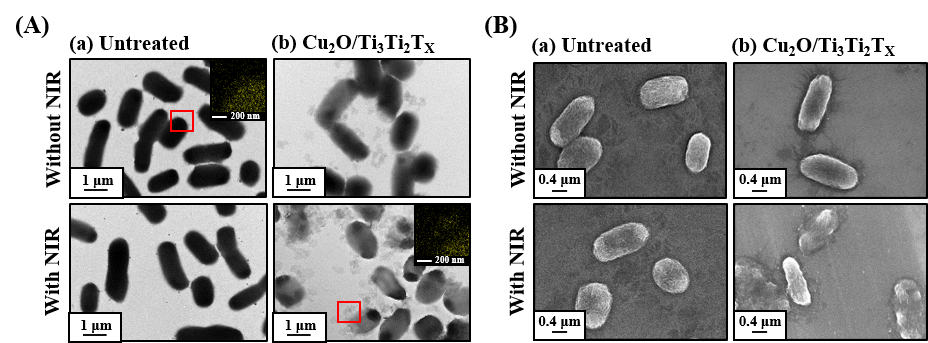


**Fig. S12.** (A) TEM and (B) SEM images of *E. coli* untreated and treated with 25 μg mL^−1^ (in terms of Ti_3_C_2_T_x_) of Cu_2_O/Ti_3_C_2_T_x_ nanosheets without or with NIR irradiation (808 nm, 0.54 W cm^−2^) for 10 min in PBS solution. The insets show the energy-dispersive X-ray spectroscopy couple elemental mapping (red square) of Cu.


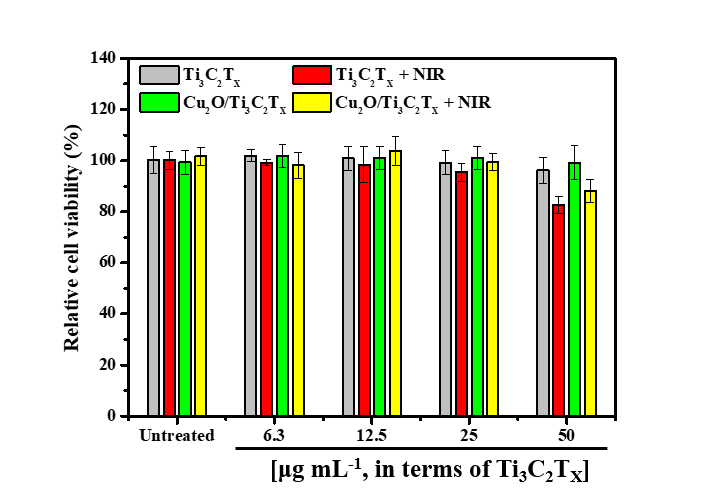


**Fig. S13.** Relative cell viability of NIH-3T3 cells untreated and treated with various concentrations (in terms of Ti_3_C_2_T_x_) of Ti_3_C_2_T_x_ or Cu_2_O/ Ti_3_C_2_T_x_ nanosheets without or with NIR irradiation (808 nm, 0.54 W cm^-2^).


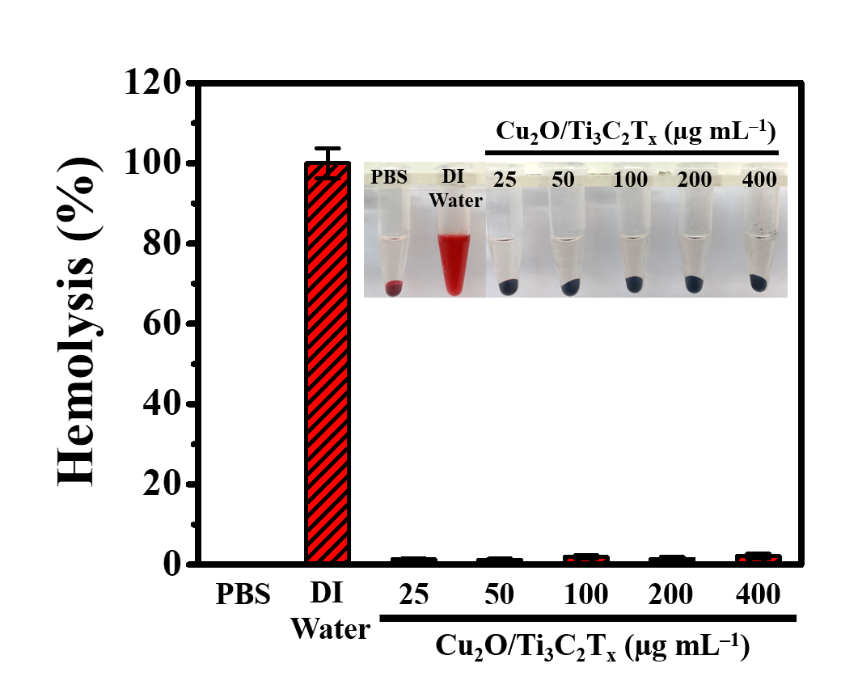


**Fig. S14.** Hemolytic activities of Cu_2_O/Ti_3_C_2_T_x_ nanosheets against RBCs dispersed in PBS. RBCs treated with PBS and DI water served as a negative and positive control groups, respectively. Insets: photographs of solutions containing RBC treated with PBS, DI water, or Cu_2_O/Ti_3_C_2_T_x_ ([Ti_3_C_2_T_x_] = 25–400 µg mL^−1^). The error bars represent the standard deviation of three repeated experiments.­


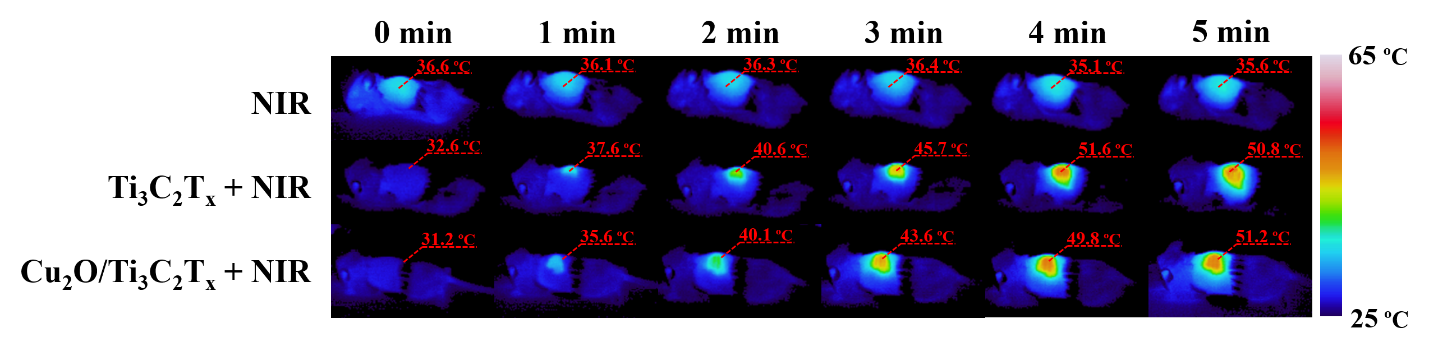


**Fig. S15.** Time-dependent thermal profiles of the mice using infrared camera under NIR irradiation (808 nm, 0.54 W cm^−2^) in the absence and presence of 50 µg mL^−1^ (in terms of MXene) of Ti_3_C_2_T_x_ or Cu_2_O/Ti_3_C_2_T_x_ nanosheets. Other conditions are same as Fig. 6.
